# Longitudinal changes in the phenotypic profile of circulating extracellular vesicles in healthy individuals

**DOI:** 10.3389/fcell.2026.1807969

**Published:** 2026-06-19

**Authors:** Kirstine Kløve-Mogensen, Rikke Bæk, Rikke W. Rasmussen, Evo K. L. Søndergaard, Aase Handberg, Maiken Mellergaard, Malene M. Jørgensen

**Affiliations:** 1 Department of Clinical Immunology, Aalborg University Hospital, Aalborg, Denmark; 2 Department of Clinical Biochemistry, Aalborg University Hospital, Aalborg, Denmark; 3 Department of Clinical Medicine, Aalborg University, Aalborg, Denmark

**Keywords:** day-to-day variation, longitudinal changes, EV Array, extracellular vesicles, high-resolution flow cytometry hFCM, phenotype

## Abstract

**Background:**

Extracellular vesicles (EVs) are emerging as valuable biomarkers in clinical diagnostics. Yet, the natural biological variation in EV concentration and phenotype among healthy individuals and across longitudinal time points remains insufficiently characterized, complicating interpretation and reproducibility in biomarker studies.

**Objective:**

To assess longitudinal (day-to-day and week-to-week) intra-individual and inter-individual variation in circulating EVs from healthy donors using complementary analytical platforms.

**Methods:**

Blood samples were collected longitudinally from four healthy female donors over 5 consecutive days and across 6 weeks. EVs were analyzed using nanoparticle tracking analysis (NTA), high-resolution flow cytometry (hFCM) targeting 3 surface markers, and the EV Array targeting 23 surface markers. Blood cell counts were also measured to explore correlations with EV profiles.

**Results:**

Significant longitudinal intra- and inter-individual variation was observed in EV concentrations and marker expression. CD9-positive EVs were consistently the most abundant across both antibody-based methods, while CD63^−^and CD81-positive EVs showed lower and more variable expression. Long-term variation exceeded short-term variation.

**Conclusion:**

This exploratory study underscores the dynamic nature of EV profiles in healthy individuals and highlights the need for standardized reference ranges and harmonized methodologies. Without accounting for biological and technical variability, EV-based biomarker studies risk misinterpretation and compromised reproducibility.

## 1 Introduction

Extracellular vesicles (EVs) are nano-sized, cell-derived particles enclosed by a lipid bilayer. These complex structures carry biological materials such as proteins, RNA, and DNA within their aqueous core (Février and Raposo, 2004; Ra et al., 2013). Traditional omics approaches have identified hundreds of proteins, lipids, and nucleic acids in EVs (Théry et al., 2002; Choi et al., 2015; Guduric-Fuchs et al., 2012; Ji et al., 2014; Hessvik et al., 2013; Simpson et al., 2008; Skotland et al., 2019). Because of their diverse content, EVs can deliver more specific and comprehensive messages than soluble molecules (Kao and Papoutsakis, 2019; Krämer-Albers and Hill, 2016). Blood is the most commonly used biological fluid in EV biomarker studies, due to its accessibility and rich informational content. EVs can be enriched from both plasma and serum using standardized protocols developed within the EV research community (Witwer et al., 2013). However, the concentration of EVs in blood remains uncertain and is heavily influenced by the chosen isolation method and potential contaminants. Blood is a complex fluid containing not only EVs but also other nano-sized particles such as lipoproteins (Sódar et al., 2016). To date, there is no reliable method for accurately quantifying blood cell-derived EVs, which poses a significant challenge to their clinical application in diagnostics and therapy (Abbott, 2023). The MISEV (Minimal Information for Studies of Extracellular Vesicles) guidelines are a comprehensive set of recommendations developed by the International Society for Extracellular Vesicles (ISEV) to standardize and improve the quality of EV research and include guidelines for flowcytometry (FCM) and nanoparticle tracking analysis (NTA) (Welsh et al., 2024). Estimates of circulating EV concentrations vary widely, from 10^7^–10^8^ EVs/mL measured by FCM to ∼10^13^ particles/mL reported by NTA. Importantly, the latter likely represents an overestimation of true EV numbers, as this technique detects all light-scattering particles within the relevant size range and therefore does not exclusively quantify EVs (Johnsen et al., 2019; Botha et al., 2021). The relationship between specific blood cell counts and circulating EV levels remains unclear. It is assumed that EV concentrations reflect a balance between secretion and uptake, but direct measurements in human plasma are lacking. Estimates suggest platelet- and erythrocyte-derived EVs occur at around 10^7^ EVs/mL, while data on leukocyte-derived EVs are limited (Arraud et al., 2014; Bettin et al., 2022; Kumar et al., 2024).

Conventional methods like Western blotting and omics analyses require EV lysis, which eliminates information about individual EVs and masks sample heterogeneity (Margolis and Sadovsky, 2019). To address this limitation, more advanced analytical techniques have been developed to study single EVs and their biological characteristics. High-resolution and imaging-based flow cytometry (hFCM) have been adapted to overcome the constraints of traditional FCM, which was designed for cells. hFCM detects fluorescence and light scatter signals from nanosize particles flowing in a stream (Welsh et al., 2023). Due to their small size and heterogeneity, some EVs remain undetectable by hFCM, whose limit of detection (LoD) is continuously improved and currently able to measure down to ∼100 nm scatter in plasma (Bettin et al., 2025). Another class of instruments, known as nano-analyzers, enables high-resolution detection of sub-micron particles. Among these, NanoFCM (nFCM) provides accurate characterization of small EVs in the 40–200 nm size range (Yim et al., 2023). Fluorescent NTA has been developed to study individual, isolated particles, but this technique is based on particle diffusion and cannot reliably assess co-expression of multiple markers (Han et al., 2021). Recent advances have introduced fluorescence-based imaging techniques such as super-resolution and confocal microscopy to investigate the expression of multiple proteins on single EVs (Chen et al., 2016; Lee W. et al., 2018; Nizamudeen et al., 2018); however, these are non-quantitative measures. Another approach involves using microarrays like the EV Array, which consists of nanoscale drops of capture antibodies printed in a well-based system. EVs are captured via their surface markers and detected using either general EV marker cocktails (CD9, CD63, CD81) or specific antibodies targeting proteins of interest (Jørgensen et al., 2013; Jørgensen et al., 2015). Additionally, several non-fluorescent technologies have been proposed to analyze individual EVs, including single-particle interferometric reflectance imaging sensing (SP-IRIS), nano-plasmonic sensors, and Raman spectroscopy (Daaboul et al., 2016; Im et al., 2014; Lee K. et al., 2018).

The aim of this study was to perform an exploratory longitudinal analysis of both long-term (week-to-week) and short-term (day-to-day) variations in EV numbers and composition in plasma from healthy individuals.

The investigations are presented as two types of variances: inter-individual variance (differences between individuals) and intra-individual longitudinal variance (changes within the same person over time).

## 2 Materials and methods

Venous peripheral blood was obtained on 5 subsequent days and in 6 subsequent weeks from four healthy (without known disease) women (age: 36–40), included as part of an exploratory pilot cohort, that were overnight fasting and provided informed consent. Blood cell counts were measured immediately, while plasma was prepared and stored for NTA, hFCM, and EV Array analysis.

### 2.1 Venous whole blood collection

Venous blood was collected using a 21-gauge needle, and the first 3.5 mL of blood was discarded. The blood was collected through venous draw into Ethylenediaminetetraacetic acid (EDTA) K3 (4 mL), or Citrate-Phosphate-Dextrose-Adenine (CPDA) (6 mL) Vacuette™ tubes (Greiner Bio-one GmbH, A). EDTA K3 samples were used directly for cell analysis. One tube of CPDA was centrifuged two times at 2,500 x g for 15 min at room temperature (RT), platelet poor plasma (PPP) was isolated and aliquoted prior to storage at −40 °C and subsequent analysis. One tube of CPDA was incubated overnight prior to centrifugation at 1,500 x g for 6 min and plasma was isolated and stored in aliquots at −40 °C (Bæk et al., 2016).

### 2.2 Whole cell analysis

Whole blood cell analysis was performed on EDTA blood on a fully automated 5-part differential haematology analyser SYSMEX XN-1000 at Aalborg University Hospital, Department of Clinical Immunology according to manufacturer’s instructions. The laboratory follows standard quality practices and is accredited by the ISO 15189 standard. Measured analytes include haemoglobin (Hb), haematocrit (Hct), red blood cell count (RBC), mean cell volume (MCV), mean cell haemoglobin concentration (MCHC), erythrocytes (ERYT), platelets (PLT), leukocytes (LEUK), lymphocytes (LYMPH), monocytes (MONO), neutrophils (NEUT), eosinophils (EO) and basophils (BASO).

### 2.3 Nanoparticle tracking analysis

NTA was conducted using a ZetaView PMX-420-Quatt instrument (Particle Metrix GmbH, DE). NTA’s laser and microscope were auto aligned using a known concentration of 100 nm polystyrene standard beads (Applied Microspheres B.V., NL). The standards and EV samples were diluted in particle-free water or phosphate-buffered saline (PBS) for analyses, respectively. For scatter mode, the particle number and size distribution were measured at 11 frames per cycle with a sensitivity of 72 and a shutter value of 100 and the ZetaView® software (version 8.05.14 SP7) was used to collect and analyse the data. Nanoparticle tracking analysis was performed directly on native, unprocessed plasma samples. No exogenous EV-mimics or spike-in controls (e.g., synthetic beads or liposomes) were added, as the study was not designed to assess EV recovery efficiency but to characterize particle-associated signals in plasma. Accordingly, NTA measurements were not intended to represent EV-specific concentrations or recovery-adjusted values.

### 2.4 High resolution flow cytometry

hFCM was performed as previously described (Rasmussen et al., 2021) with full detailed descriptions presented in supplementary material, adhering to MISEV2023 and MIFlowCyt-EV guidelines (Supplementary Table S1, S2) (Welsh et al., 2024; Welsh et al., 2020).

Specifically, PPP samples were thawed and stained with antibody mix solutions. Antibodies used for analysis were FITC-CD9 (HI9a; Biolegend, CA, US), PE-CD81 (5A6; Biolegend, CA, US), APC-CD63 (H5C6; Biolegend, CA, US), and matched isotype controls: IgG1κ (MOPC-21; FITC, PE, and APC; Biolegend, CA, US). Isotype controls were used to assess background fluorescence but were not applied as fluorescence-minus-one (FMO) controls for refined gating close to the limit of detection. Antibody and PBS (Merck, DE, Cat. D8537) mixes were filtered through 0.45 μm filters. Ten µL of PPP was stained directly with 50 µL of master or isotype mix solution, incubated for 2 h at RT in the dark. Next, samples were diluted individually in PBS to obtain a flow rate of 2000–6000 events/sec. Diluted samples were kept at RT and dark until analysis. Potential aggregates in the antibody and isotype mix solutions were analyzed in PBS. Lastly, detergent lysis control was included for all stained samples to verify the staining of EV populations by incubating stained sample for at least 30 min at RT in the dark with 1% v/v Triton X-100 (Merck, DE, Cat. 93443). Analysis was done using an Apogee A60Micro-PLUS high-resolution flow cytometer (Apogee Flow Systems, UK) with three diode lasers, with settings described in Supplementary Table S3, data acquisition was done at 3.01 μL/min for 180 s with a triggering threshold value at 27 (1728) on medium angle light scatter (MALS). Cleaning between each sample was done with Apogee Clean (Apogee Flow Systems, United Kingdom, Cat 1519) and PBS at flow rate of 10.5 μL/min for 180 s (cleaning was repeated if necessary, maintaining the background levels <100 events/sec for PBS between samples). Data were recorded in the Histogram software (version 6.0.72, Apogee Flow Systems, United Kingdom). Daily performance of the hFCM was ensured for volumetric quantification (Supplementary Table S2 section 4.2) and tested using beads. Fluorescence was calibrated (MESF, Supplementary Figure S2). Compensation was done using Spherotech compensation beads (Spherotech, IL, United States, Cat. CMIg-08-2K; compensation matrix in Supplementary Figure S3).

Raw flow cytometry signals were analyzed using the FlowJo software version 10.10.0 (BD Biosciences, CA, United States). Gating was done as shown in Supplementary Figure S4. Firstly, samples were calibrated using the Rosetta Calibration software v2.05 (Exometry, NL) and a 0–1000 nm gate was applied to all samples, thereby restricting analyses to the detectable nanoparticle fraction under the applied instrument settings. Positive events were defined according to the isotype background and EV concentrations were calculated as exemplified: EVs/mL = ((CD9^+^-positive EVs)/(volume of the run sample (pL))) * sample dilution factor * 1.000.000.

### 2.5 EV Array

During production of the microarrays, antibodies were printed on epoxy-coated slides (75.6–25.0 mm; SCHOTT Nexterion, DE) using a sciFLEXARRAYER S12 installed with a PDC60 with coating type 3 (Scienion AG, DE). Biotinylated goat anti-mouse immunoglobulin G (20, 10 and 5 μg/mL; Novus Biologicals, CO, US) was used as positive controls and PBS with 50 mM trehalose was used as negative control. After the printing procedure, the slides were left to dry at RT overnight before further use. Twenty-three anti-human antibodies were printed (with the corresponding clone, if available): LFA-1 (HI111; Ab biotec, CA, US); Flotillin-1 (Abcam, MA, US); CD9 (SN4/C3-3A2), CD81 (1.3.3.22; Ancell, MN, US); CD3 (Hit3a), CD56 (3G8; BD Biosciences, NJ, US); Alix (3A9), HLA ABC (W8/32; Biolegend, CA, US); CD63 (Mem-259; BioRad, CA, US); HLA DR/DP/DQ (HB-145/IVA12; Caprico Biotechnologies, GA, US); ICAM-1 (R6.5; eBiosciences, CA, US); CD42a, CTLA4 (ANC152.2/8H5; LifeSpan BioSciences, WA, US); CD106 (HAE-2Z), CD142 (323514), CD31, CD4 (34930), CD45 (2D1), CD80 (37711), CD8a (37006), MIC A/B (159207), TNF RI (16803), TNF RII (22210; R and D Systems, MN, US). All antibodies were diluted in PBS with 50 mM trehalose and printed in triplicates at 200 μg/mL.

The EV Array analysis was performed as described by Jørgensen et al. (2013), Jørgensen et al. (2015) with minor modifications. In short, the microarray slides were blocked (50 mM ethanolamine, 100 mM Tris, and 0.1% sodium dodecyl sulfate, pH 9.0) before incubation with samples diluted (1:5) in incubation buffer (0.5X Casein (Sigma-Aldrich, MO, US), 0.1% Tween 20® in PBS). The microarray slides were incubated in Multi-Well Hybridization Cassettes (ArrayIt Corporation, CA, US) at RT for 2 h followed by overnight incubation at 4 °C. After a wash in washing buffer (0.05% Tween 20® in PBS), slides were incubated with biotinylated detection antibodies as either a cocktail or singular (anti-human CD9 (SN4/C3-3A2), anti-human CD63 (AHN16.1/46-4-5), and/or anti-human CD81 (1.3.3.22), LifeSpan BioSciences, WA, US) diluted 1:1,500 in incubation buffer for 2 h. After additional washing, incubation for 30 min with cyanine 5–labeled streptavidin (Life Technologies, CA, US) diluted 1:1,500 in incubation buffer was carried out for detection. Before scanning, slides were washed in wash buffer followed by MilliQ water and dried using a Microarray High-Speed Centrifuge (ArrayIt Corporation, CA, US). Scanning and spot detection were performed as previously described (Jørgensen et al., 2013; Jørgensen et al., 2015).

Co-localization for the EV Array was calculated as the mean intensity of EV signals obtained from two reciprocal capture–detection pairs. For example, for CD9×CD81, the mean was taken between the intensity of EVs captured with CD9 and detected with CD81, and EVs captured with CD81 and detected with CD9.

### 2.6 Statistical analysis

All analytical measurements are presented as raw data. Data visualization was performed using XY scatter plots and heatmaps to illustrate correlations and distribution patterns. To assess the relative variability of the measured parameters, the coefficient of variation (CV) was calculated. CV values exceeding 15% were considered indicative of high variability, unless otherwise justified by biological or technical factors. Variance component considerations were approximated based on observed CV and typical analytical CV ranges (5%–20%), assuming additive contributions of technical and biological variance. Spearman rank correlation was used to assess monotonic relationships between variables, and the resulting correlation matrices display the Spearman correlation coefficients (ρ) for each variable pair. Correlations between 0.40–0.59 were considered moderate, 0.60–0.79 strong, and correlation between 0.80–1.00 very strong. Calculations were performed using Microsoft Excel and analyses were performed using GraphPad Prism 10.4.1 (GraphPad Software Inc., CA, US) software. Short-term and long-term variability were assessed using linear mixed-effects models implemented in R (version 4.5.2). For both day- and week-resolved datasets, log-transformed values were modelled separately for each analyte with analytical method included as a fixed effect and timepoint included as a random intercept to capture intra-individual temporal variability. Due to the limited number of donors, inter-individual variance could not be robustly modelled as a separate random effect and is therefore captured together with technical variability in the residual term. Models were fitted using restricted maximum likelihood. Variance components attributable to timepoint and residual error were extracted from each model and visualised as absolute and relative contributions using stacked bar plots. All analyses were performed using the R packages lme4 and lmerTest for model fitting and inference, dplyr for data handling, ggplot2 for visualisation, readxl for data import, and patchwork for multi-panel figure assembly.

## 3 Results

### 3.1 Intra-individual variation

#### 3.1.1 Concentrations

**FIGURE 1:**
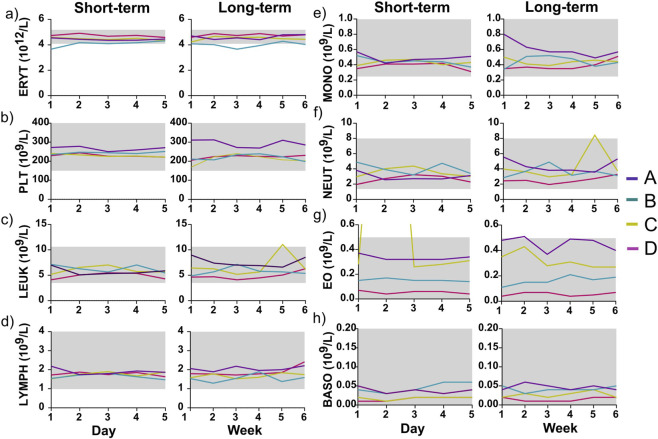
Short- and long-term intra-individual variability in blood cell counts for four donors **(A–D)**, including: **(a)** erythrocytes (ERYT), **(b)** platelets (PLT), **(c)** leukocytes (LEUK), **(d)** lymphocytes (LYMPH), **(e)** monocytes (MONO), **(f)** neutrophils (NEUT), **(g)** eosinophils (EO), and **(h)** basophils (BASO). *Grey areas are defined normal areas (ERYT: 4.2–5.4 × 10^12^/L; PLT: 150–400 × 10^9^/L; LEUK: 4.5–11.0 × 10^9^/L; MONO: 0.2–1.0 × 10^9^/L; NEUT: 1.5–8.0 × 10^9^/L; LYMPH: 1.0–4.0 × 10^9^/L; EO: <0.5 × 10^9^/L; BASO: <0.2x10^9^/L).

**FIGURE 2:**
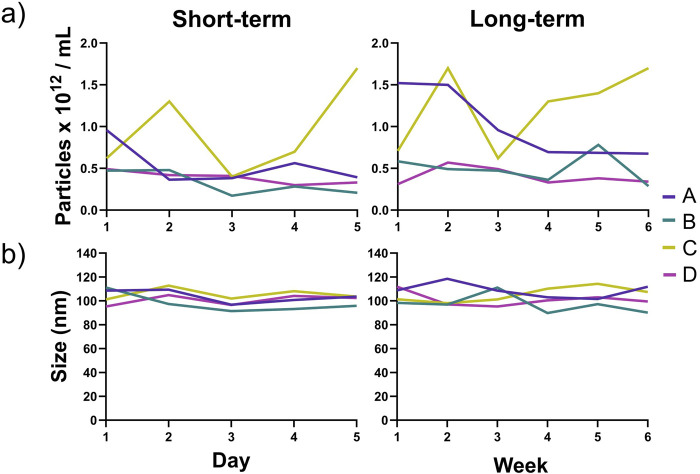
Temporal stability of four donors (A–D) measured with nanoparticle tracking analysis (NTA) showing: **(a)** particle concentration and **(b)** estimated particle size.

**FIGURE 3:**
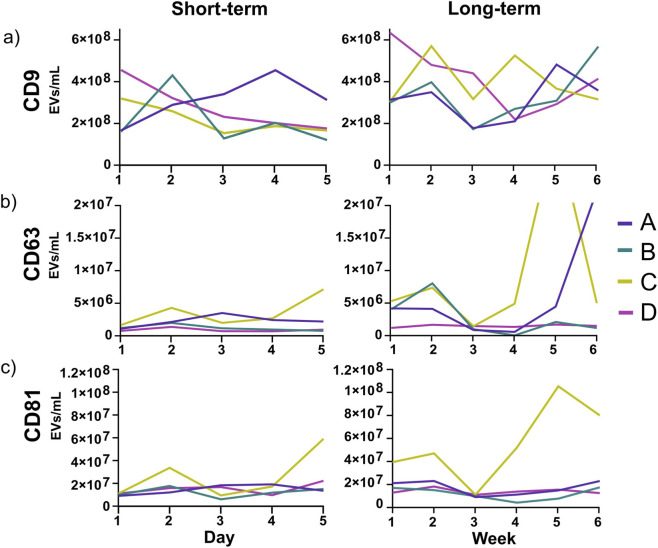
Comparison of short- and long-term measurements by high-resolution flow cytometry (hFCM) for four donors (A–D), illustrating: **(a)** CD9^+^-EVs, **(b)** CD63^+^-EVs, and **(c)** CD81^+^-EVs.

**FIGURE 4:**
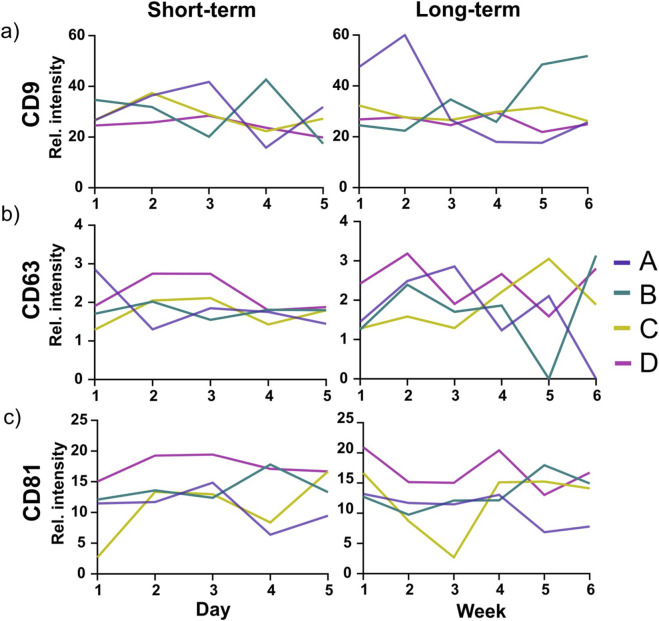
Longitudinal assessment of four donors (A–D) with EV Array-derived relative signal intensities for covering: **(a)** CD9^+^-EVs, **(b)** CD63^+^-EVs, and **(c)** CD81^+^-EVs.

**FIGURE 5:**
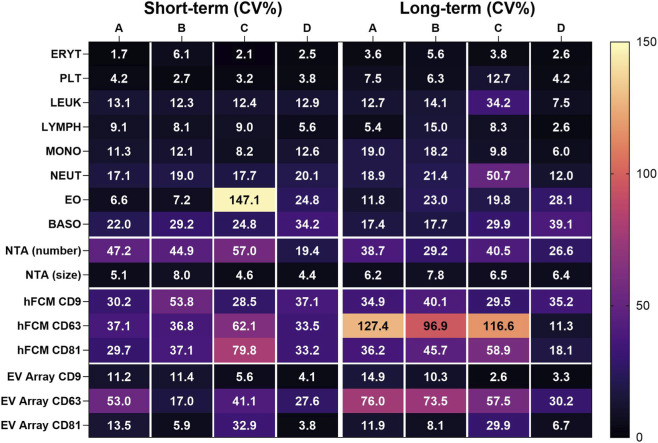
Coefficient of variance (CV) of short- and long-term intra-individual comparison in measurements of four donors (A–D) for blood cell counts, nanoparticle tracking analysis (NTA)-determined particle number and size, and CD9^+^-, CD63^+^-, and CD81^+^-EVs with high-resolution flow cytometry (hFCM) and EV Array.

**FIGURE 6:**
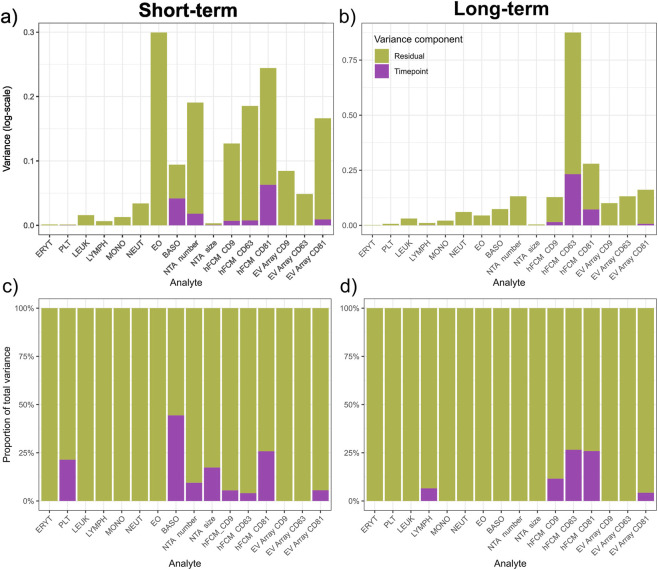
Stacked bar plots show variance components estimated using linear mixed-effects models for blood cell counts, NTA-derived particle number and size, and CD9^+^, CD63^+^, and CD81^+^ EVs measured by high-resolution flow cytometry (hFCM) and EV Array. Absolute variance on the log scale is shown for short-term **(a)**; 5 consecutive days and long-term **(b)**; 6 weeks measurements. Panels **(c)** and **(d)** show the relative contribution of variance components. Variance is partitioned into intra-individual temporal variability timepoint (purple) and residual variance (green).

The intra-individual variation was measured over 6 weeks with one sample per week (long-term), and additionally with one sample every day for 5 days in the same week (week 3) (short-term). On Figure 1 the cell counts of erythrocytes (ERYT), platelets (PLT), leukocytes (LEUK), lymphocytes (LYMPH), monocytes (MONO), neutrophils (NEUT), eosinophils (EO) and basophils (BASO) at all time points for the four donors (A-D) are presented. Normal cell count areas are indicated according to (Brihi and Pathak, 2024). Individual measurement can be seen in Supplementary Table S4. Figure 2 shows NTA measurements of particles numbers and predicted particle size. On Figures 3, 4 measurements of CD9^+^-, CD63^+^-, and CD81^+^-EVs at different timepoints with two different methods, hFCM and EV Array, are presented. CVs were calculated for both short- and long-term measurements for the four donors for each of the measured parameters, as presented in Figure 5. Linear mixed-effects models (Figure 6; Supplementary Table S9) revealed that, for most analytes, variability was dominated by residual variance, whereas intra-individual temporal variability was small or absent across both short-term (day-to-day) and long-term (week-to-week) measurements. CD9^+^- and CD81^+^-EVs were measured in the highest quantities with both methods. Regarding the cell counts this was predominantly observed for granulocyte subtypes. NEUT CVs were for all donors, except one, above 15% and up to 50.7%. For EO three out of four donors revealed CVs with high variability, on one occasion up to 147.1%. The BASO cell counts exceeded 15% for all four donors at both short- and long-term with CVs up to 39.1%. On one occasion high variability was observed for LEUK (CV: 34.2%) and for two for MONO (CV: 18.2% and 19.0%). The high CVs observed in basophils and eosinophils, probably reflect their low abundance and inherent biological fluctuation. These findings suggest that while some cell type counts maintain relatively stable over time, others—particularly granulocyte subtypes—exhibit significant variation that may impact diagnostic reliability and longitudinal monitoring.

NTA measurements of particle concentration (number) showed high variability (CV: 19.4%–57.0%) both at short- and long-term measurements, while the NTA size prediction was more stable (Figures 2, 5). These results highlight that while NTA provides relatively stable size measurements, particle concentration estimates are subject to considerable fluctuation, which may reflect both technical limitations and sample heterogeneity.

The presence of CD9^+^-, CD63^+^, and CD81^+^-EVs in the samples were quantified with two different techniques, hFCM and EV Array. High variability was more pronounced for hFCM, where CVs for CD9^+^-EVs ranged from 28.5%–53.8%, CD63^+^-EVs: 11.3%–127.4%, and for CD81^+^-EVs: 18.1%–58.9%. For EV Array, high variability was almost limited to the presence of CD63^+^-EVs, which according to both hFCM and EV Array, Figures 3, 4, are the surface biomarkers that are detected in the smallest amounts. Although the intensity of individual EV markers measured with EV Array did not change significantly between samples, either in the short- or long-term, there were differences among the four donors, Supplementary Figure S5. Separate analysis with the three detection antibodies alone showed the highest similarity between the cocktail and CD9, displaying a nearly identical pattern of detected EV markers. Use of CD63 as detection antibody resulted in very low intensities, and only signal for CD9 and CD42 were present, see Supplementary Figure S6. Similarly, CD81 detected only a few markers, notably CD9, CD42a, and CD81. CD9 demonstrated the most consistent performance over time, while CD63 showed substantial variability. These results indicate that CD9 and CD81 provide more stable signals in both short- and long-term settings, whereas CD63 may be more sensitive. This correlates with consistently lower signal intensities measured for CD63, making it more sensitive to even minor fluctuations (Figures 4B, 5).

#### 3.1.2 EV phenotypes and co-localization

**FIGURE 7:**
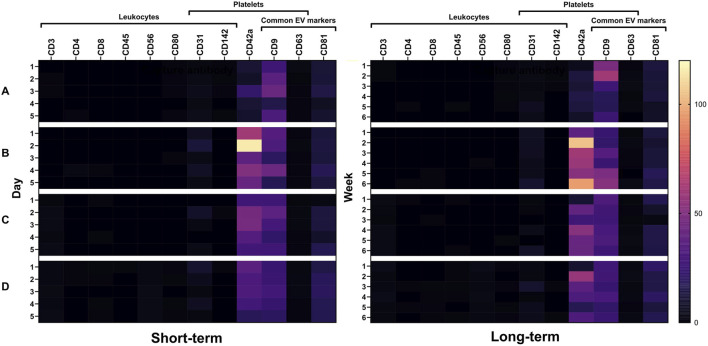
Short- and long-term relative intensities of 12 surface antibodies measured for four donors (A–D) with EV Array (detected with a cocktail of biotinylated CD9, CD63 and CD81) and presented as heatmaps.

**FIGURE 8:**
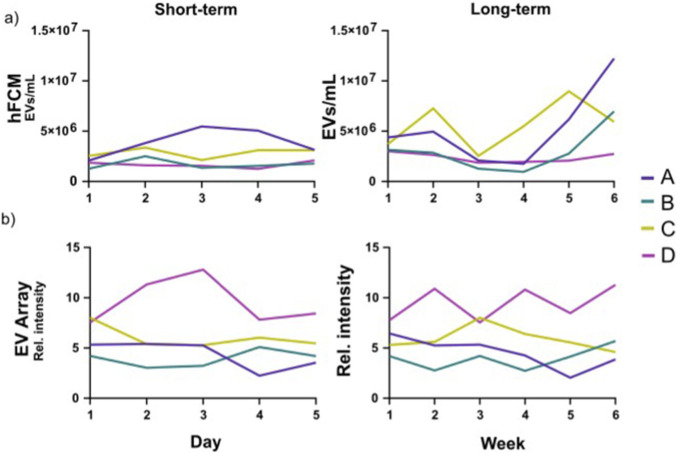
Short- and long-term determinations of co-localization of CD9 and CD81 on EVs (CD9^+^CD81^+^-EVs) determined with **(a)** high-resolution flow cytometry (hFCM) and **(b)** EV Array.

Measured intensities of 12 of the 23 investigated surface antibodies are presented as a heatmap on Figure 7, and the presence of all 23 surface antibodies are shown in Supplementary Figure S5. This analysis revealed the highest presence of platelet-related antibodies (CD9, CD31 and CD42a) and the common EV markers CD9 and CD81. Co-localization of CD9, CD63 and CD81 on the surface of EVs was quantified with the use of both hFCM and EV Array. The co-localization of CD9 and CD81 (CD9^+^CD81^+^-EVs) was by far the most dominant and identified 10-fold more frequently than CD9^+^CD63^+^-EVs by both methods. CD81^+^CD63^+^-EVs were rarely detected. The short- and long-term measurements of CD9^+^CD81^+^-EVs in the four donors are presented in Figure 8. CD81^+^CD63^+^-EVs and CD81^+^CD63^+^-EVs are shown in Supplementary Table S7 and S8. Short-term CV for hFCM ranged from 17.6%–35.4%, while long-term ranged from 20.1%–72.4%. For EV Array, the short-term CV ranged from 19.0%–32.5%, and the long-term from 18.1%–33.5%.

### 3.1.3 Correlations between cell types and EV Array

**FIGURE 9:**
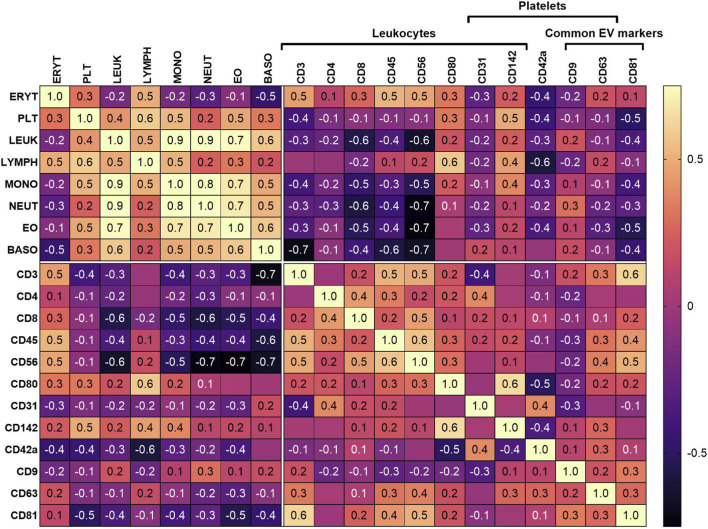
Correlation matrix between blood cell counts (erythrocytes (ERYT), platelets (PLT), leukocytes (LEUK), lymphocytes (LYMPH), monocytes (MONO), neutrophils (NEUT), eosinophils (EO) and basophils (BASO)) and relative intensities of 12 surface antibodies measured with EV Array (detected with a cocktail of biotinylated CD9, CD63 and CD81). Correlations are calculated on long-term measurements of donor A-D as Spearman correlations coefficients.

Spearman correlation of cell counts and EV Array data (Figure 9) revealed moderate to strong correlations for several cell types. ERYT were moderately positively correlated with CD3, CD45 and CD56 (ρ = 0.5), and moderately negatively correlated with CD42a (ρ = −0.4). PLT were moderately positively correlated with CD142 (ρ = 0.5), and moderately negatively correlated with CD42a and CD81 (ρ = −0.50 to −0.40). LEUK were strongly negatively correlated with CD8 and CD56 (ρ = −0.6). LYMPH were strongly positively correlated with CD80 (ρ = 0.6) and moderately positively correlated with CD142 (ρ = 0.4). MONO were moderately negatively correlated with CD8 and CD56 (ρ = −0.5). NEUT were moderately negatively correlated with CD45 (ρ = −0.4) and strongly negatively correlated with CD8 (ρ = −0.6) and CD56 (ρ = −0.7). EO were moderately negatively correlated with CD8 and CD81 (ρ = −0.5), and strongly negatively correlated with CD56 (ρ = −0.7). BASO were moderately negatively correlated with CD4 and CD81 (ρ = −0.4), and strongly negatively correlated with CD3 (ρ = −0.7), CD45 (ρ = −0.6) and CD56 (ρ = −0.7).

Several moderate correlations were observed among EVs with the markers such as CD3×CD46, CD3×CD56, CD3×CD31, CD4×CD8, CD4×CD31, CD8×CD56, CD45×CD81, CD56×CD63, CD80×CD42a, CD31×CD42a, and CD142×CD42a. In contrast, strong correlations were found for CD3×CD81, CD45×CD56, and CD80×CD142.

### 3.2 Inter-individual variation

**FIGURE 10:**
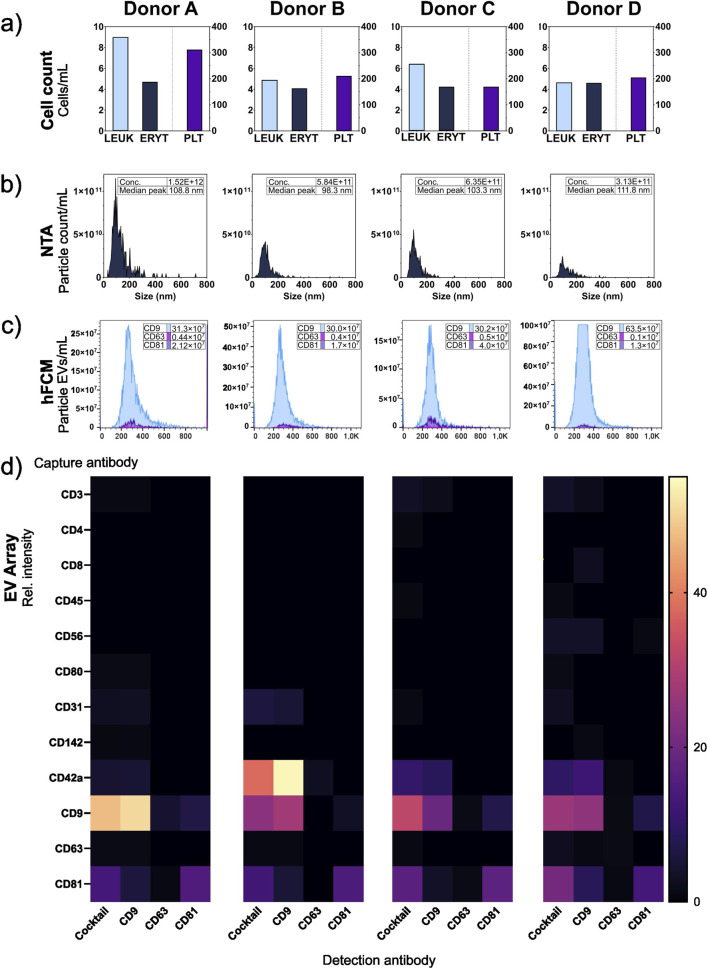
Inter-individual comparison of the four donors (A-D) at one time point (week 1, day 1) of **(a)** blood cell counts of leukocytes (LEUK), erythrocytes (ERYT) and platelets (PLT), **(b)** particle counts with nanoparticle tracking analysis (NTA), **(c)** counts of CD9^+^-, CD63^+^-, and CD81^+^-EVs with high-resolution flow cytometry (hFCM), and **(d)** relative intensity measures of 12 surface antibodies with EV Array (detected with biotinylated CD9, CD63, CD81 or a cocktail consisting of all three).

**FIGURE 11:**
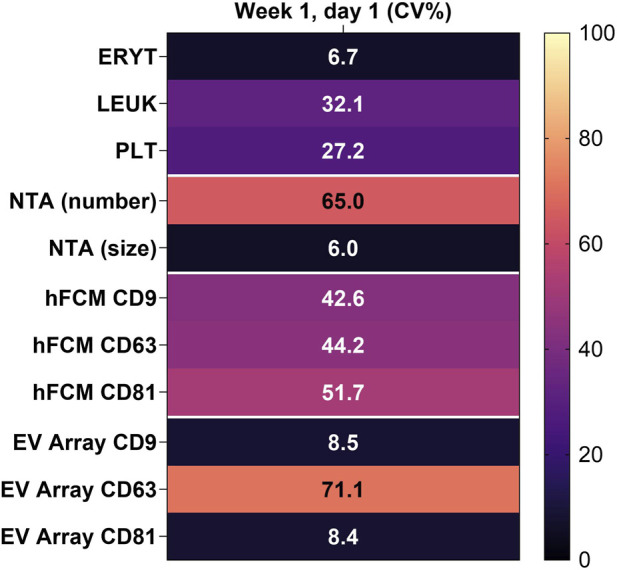
Inter-individual coefficient of variation calculated for the four donors (A–D) at one time point (week 1, day 1) of blood cell counts of erythrocytes (ERYT), leukocytes (LEUK) and platelets (PLT), particle counts with nanoparticle tracking analysis (NTA), positive CD9^+^-, CD63^+^-, and CD81^+^-EVs with high-resolution flow cytometry (hFCM) and intensities of CD9^+^-, CD63^+^-, and CD81^+^-EVs with EV Array (detected with a cocktail of CD9, CD63 and CD81).

The study also revealed a high degree of inter-individual variation. This is highlighted in Figure 10, which compared the results for all four donors at a single time point (week 1, day 1). Inter-individual variability was also assessed by calculating the CV across the four healthy donors measured at the same time, Figure 11. Among cellular components, LEUK showed moderate variability (CV: 32.1%), while ERYT was highly consistent (CV: 6.7%). PLT exhibited intermediate variation (CV: 27.2%). For NTA, particle number demonstrated substantial variability (CV: 65.0%), whereas size was highly stable (CV: 6.0%). EV characterization by hFCM revealed CVs of 42.6% for CD9^+^-EVs, 44.2% for CD63^+^-EVs, and 51.7% for CD81^+^-EVs in the 0–1000 nm range, indicating moderate to high variability between individuals. EV Array analysis showed low variability for CD9 (CV: 8.5%) and CD81 (CV: 8.4%), but markedly higher variation for CD63 (CV: 71.1%).

These findings suggest that inter-individual differences are most pronounced in EV-related measurements, particularly for CD63 detected by EV Array and particle number by NTA. In contrast, ERYT counts and NTA size measurements were highly consistent across donors. This indicates that while some parameters (e.g., ERYT counts, EV Array CD9/CD81) are robust across healthy individuals, others (e.g., EV Array measured CD63, NTA particle number) may be strongly influenced by biological variability, which should be considered when defining reference ranges or standardizing assays.

## 4 Discussion

Despite growing interest in blood-derived EVs as biomarkers, substantial knowledge gaps remain. A key limitation is the absence of an accepted baseline for EV concentrations (Johnsen et al., 2019). EV levels are influenced by clinical status, demographic factors, lifestyle, and longitudinal biological variation among healthy individuals. A deeper understanding of this inherent variability is essential for interpreting EV based diagnostic data. While EV isolation strategies such as SEC improve vesicle specificity, they also alter the native particle composition of plasma; the present study therefore prioritizes physiological relevance over vesicle purity and does not aim to quantify absolute EV recovery efficiency. Our observations align with earlier studies reporting greater inter-individual than intra-individual variation in EV concentrations (Aziz et al., 2019; Widjaja et al., 1999; Holcar et al., 2025). Although the diversity of EV subtypes is well recognized, current analytical methods rarely offer sufficient resolution to quantify EV subpopulations reliably. As a result, important questions—such as which EV subsets carry distinct biological functions or diagnostic value—remain unanswered. While emerging technologies are beginning to address these limitations, their use is still not widespread.

In this exploratory longitudinal study, we evaluated both short- and long-term CVs across several analytical platforms. Among blood cell populations, reproducibility was generally high. ERYTs and PLTs showed low variability (short term CVs <6.5%; long term <13%). In contrast, granulocyte subsets displayed high variability, particularly in short term measurements, likely due to low cell counts or intrinsic biological variation. NEUTs also showed elevated long-term variability (up to 50.7%), potentially reflecting biological fluctuations or sensitivity to preanalytical conditions. These findings support the robustness of measurements for major leukocyte subsets (LEUK, LYMPH, MONO), while caution is warranted for rarer cell populations.

NTA revealed substantial longitudinal variability in particle concentration (short term CVs: 19.4%–57.0%; long term: 26.6%–40.5%), reflecting the combined influence of technical and biological variation. Because the NTA measurements were label free, EVs could not be distinguished from other colloidal particles of the systemic circulation, such as lipoproteins. Lipoproteins consistently fluctuate in response to dietary modifications and are present in plasma with 10^5^–10^6^ fold higher abundance than EVs (Johnsen et al., 2019; Cohn et al., 1988). Lipoproteins are known contaminants in EV isolates and even carry biomarker molecules themselves. In contrast to concentration, NTA particle size measurements were considerably more stable (short term CVs: 4.4%–8.0%; long term: 6.2%–7.8%), confirming that NTA is reliable for size profiling, though less so for quantitative assessment. The observed variability in particle concentration may in part reflect size-dependent detection effects inherent to NTA. Specifically, inclusion of very small particles (e.g., protein aggregates or lipoproteins) or larger non-EV particles may introduce variability due to differences in scattering intensity and detection sensitivity. Applying size-restricted analysis or excluding particles below a defined threshold may reduce such variability and improve robustness of concentration measurements.

hFCM analysis showed substantial variability across tetraspanin markers. CD9^+^- and CD81^+^-EVs displayed moderate reproducibility (short term CVs: 28.5%–79.8%; long term: 18.1%–58.9%), whereas CD63 showed the highest variability, with some long-term CVs exceeding 100%. This likely reflects low signal intensity and high susceptibility to technical fluctuations, limiting the utility of CD63 as a quantitative marker, at least for the sample material and the LoD of the analyses utilized in the current study.

Consistent with earlier findings that ∼49% of tetraspanin signals co-localize (Han et al., 2021), our hFCM and EV Array analyses showed that CD9^+^CD81^+^-EVs were the most prevalent. This supports findings from other studies showing similar double-positive patterns (Han et al., 2021). Additionally, research using hFCM has shown co-occurrence of CD9 and CD81 into HIV-1 particles (Jeong et al., 2018) and a correlation between co-expression and vesicle size (Dahmane et al., 2019). CD63^+^-EVs, however, were predominantly CD63 single positive, and when co-localized, they tended to form CD9×CD63×CD81 triple positive populations (Barranco et al., 2019). Although there is no clear consensus on how tetraspanins are distributed across EV subtypes, several studies suggest that CD9 and CD81 are more commonly associated with microvesicles, whereas CD63 may be enriched in exosomes (Han et al., 2021; Han et al., 2023). This could explain the low signals observed in the EV Array and is consistent with the current limitation of hFCM, which has difficulty detecting particles smaller than 100 nm, and should be interpreted in light of size-dependent detection limits and background thresholding strategies.

Compared with hFCM, the EV Array demonstrated notably higher longitudinal reproducibility, especially for CD9 and CD81. Short-term CVs for CD9 were 4.14%–11.4%, and long-term CVs 2.6%–14.9%; CD81 showed similar patterns. CD63 again displayed higher variability, though still lower than for hFCM. These results highlight the importance of analytical platforms, marker selection, and gating strategies. CD9 and CD81 appear to provide more stable readouts, whereas CD63 remains technically challenging, likely related to the low expression/numbers observed. Further standardization and methodological optimization along with more knowledge on the regulation and expression patterns of the tetraspanins are critical for advancing EV based diagnostics.

Across all measured blood cell measurements, long-term variability exceeded short-term variability, except for EO. NTA and hFCM also showed higher long-term than short-term variability, driven largely by CD63 in the case of hFCM. EV Array measurements remained comparatively stable in longitudinal assessments, with only modest long-term increases, again attributable to CD63. Spearman correlation analysis revealed several biologically plausible associations between blood cell counts and EV surface markers. PLT, which includes both active and resting platelets, correlated positively with CD142 (tissue factor) intensities. This association may reflect the role of P-selectin on activated platelets in triggering CD142 exposure on monocytes, a mechanism proposed to promote rapid intravascular coagulation (Ivanov et al., 2019). CD142 is expressed by 20%–30% of resting platelets and translocates to the surface upon activation, where it plays a central role in coagulation (Brambilla et al., 2024). Furthermore, CD142 has previously been linked to circulating microparticles and EVs (Zwicker et al., 2011; Dignat-George and Boulanger, 2011). PLTs showed a negative correlation with CD42a (GPIX) expression on EVs, a key surface glycoprotein involved in platelet adhesion and clot formation (Keller Cecconello et al., 2024).

The negative correlation could suggest that as platelet numbers rise, fewer EVs express CD42a, potentially reflecting reduced vesiculation or activation per platelet, or a phenotypic shift in EV populations during states of increased platelet turnover; however, this interpretation remains exploratory.

LYMPH counts correlated positively with CD80, an “immunologically important” molecule enriched on B cell derived exosomes (Clayton et al., 2001). This positive correlation likely reflects that higher lymphocyte counts include more B cells, which release CD80-enriched EVs as part of immune signaling.

Inter-individual comparisons across the four healthy donors showed marked biological differences in EV and cellular parameters. ERYT counts and NTA based size measurements were highly stable (CVs: 6.0–6.7), whereas NTA particle counts (CV: 65.0) and hFCM measured tetraspanins (CD9, CD63, CD81) showed moderate variability, indicating differences in EV abundance and marker expression between individuals. EV Array measurements for CD9 and CD81 were highly consistent, supporting their suitability as robust markers. CD63 again showed poor reproducibility, consistent with earlier studies. These results emphasize the importance of selecting stable markers and accounting for individual variability when developing EV based assays.

Overall, our exploratory data demonstrates substantial longitudinal intra- and inter-individual variability in EV concentrations and phenotypes among healthy individuals. Across all analytical methods (NTA, hFCM, EV Array), EV/particle counts, and marker expression fluctuated markedly over day-to-day and week-to-week intervals, particularly for CD9 and CD81. These fluctuations mirrored changes in blood cell counts, especially platelets. Considering this, determining the concentrations of platelet-derived EVs in the samples by hFCM would shed light on their possible impact on the variation of EVs in the samples, although this was out of scope of the current study.

Variance component analysis demonstrated that for most analytes, only a minor proportion of total variance could be attributed to systematic timepoint effects, whereas the majority of variability was captured in the residual term, which encompasses both technical variability and inter-individual biological heterogeneity that could not be robustly modeled separately due to the limited cohort size.

To estimate cohort size requirements for future studies aiming to disentangle technical and biological variability, we used the observed CVs across analytes, assuming typical analytical CVs of 5%–20%. Low-variability parameters such as ERYT and PLT (CV <10%) likely reflect comparable technical and biological contributions, whereas leukocyte subsets (e.g., NEUT, MONO) and EV-related measurements (NTA, hFCM CD9/CD63/CD81, EV Array) showed substantially higher CVs (>20–50%), indicating dominant biological heterogeneity. Based on these observations, we estimate that >80–100 subjects are required for low-variability analytes, while 30–60 subjects are sufficient for high-variability markers. These estimates may serve as a practical reference for study design in future EV biomarker investigations.

Altered EV profiles have been linked to a wide range of diseases, including cardiovascular disease (Nomura et al., 2003), infections (Joop et al., 2001), and autoimmune diseases such as multiple sclerosis (Sheremata et al., 2008), rheumatoid arthritis (Knijff-Dutmer et al., 2002), and systemic lupus erythematosus (Østergaard et al., 2013; Nielsen et al., 2012), as well as numerous cancers (Janowska-Wieczorek et al., 2006). Disease association studies frequently compare patient samples with healthy controls; however, our results highlight the risks of this approach in the absence of baseline values. Because EV concentrations in healthy individuals vary over time, differences between patients and controls may reflect timing rather than disease, increasing the risk of false positive biomarker identification (Krzyslak et al., 2026). This highlights the need for control groups of considerable size, as larger groups help smooth out variability and stabilize the signal. Our findings reinforce the need for additional research before EVs can be confidently used as disease specific biomarkers.

The strengths of this study include the use of several analytical platforms and the many time points, while a limitation is the small number of individuals investigated. The results presented in this paper represent the total variance, encompassing longitudinal biological variation as well as pre-analytical factors such as technical, analytical, and sample handling differences. The variance is presented as a total and represents variance in measurements of samples taken at the same time from the same donors, handled and analyzed by the same personnel in the same laboratory setups, limiting these factors to as little as possible.

A limitation of the present study is the absence of exogenous EV-mimics or spike-in controls to assess absolute EV recovery efficiency. While such controls are highly relevant in studies focusing on EV isolation or recovery, the deliberate aim here was to analyze particles directly in native plasma without altering the physiological particle composition. Future studies combining native plasma analysis with controlled spike-in approaches may provide complementary insights.

A major limitation in the field is the lack of standardized reference materials that can be utilized across different methods—such as artificial or purified EV preparations with defined particle counts—which is essential for instrument calibration and inter laboratory comparability. Addressing this gap is crucial for improving reproducibility and advancing EV based research and clinical diagnostics.

In conclusion, the absence of a standardized EV baseline, combined with substantial biological variability, poses a major challenge for disease association studies. Establishing reference ranges, improving standardization of pre-analytical and analytical workflows, and developing technologies capable of resolving EV subpopulations with high precision will be essential for translating EV based biomarkers into clinical practice. Until such standards are in place, EV based biomarker data must be interpreted with caution.

## Data Availability

The datasets presented in this study can be found in online repositories. The names of the repository/repositories and accession number(s) can be found in the article/Supplementary Material.

## References

[B1] Abbott A. (2023). FedEx for your cells: this biological delivery service could treat disease. Nature 621 (7979), 462–464. 10.1038/d41586-023-02906-w

[B2] Arraud N. Linares R. Tan S. Gounou C. Pasquet J. M. Mornet S. (2014). Extracellular vesicles from blood plasma: determination of their morphology, size, phenotype and concentration. J. Thromb. Haemost. 12 (5), 614–627. 10.1111/jth.12554 24618123

[B3] Aziz N. Detels R. Quint J. J. Gjertson D. Ryner T. Butch A. W. (2019). Biological variation of immunological blood biomarkers in healthy individuals and quality goals for biomarker tests. BMC Immunol. 20 (1), 33. 10.1186/s12865-019-0313-0 31521107 PMC6744707

[B59] Bæk R. Søndergaard E. K. Varming K. Jørgensen M. M. (2016). The impact of various preanalytical treatments on the phenotype of small extracellular vesicles in blood analyzed by protein microarray. J Immunol Methods. 438, 11–20. 10.1016/j.jim.2016.08.007 27568281

[B4] Barranco I. Padilla L. Parrilla I. Álvarez-Barrientos A. Pérez-Patiño C. Peña F. J. (2019). Extracellular vesicles isolated from porcine seminal plasma exhibit different tetraspanin expression profiles. Sci. Rep. 9 (1), 1–9. 10.1038/s41598-019-48095-3 31399634 PMC6689046

[B5] Bettin B. Gasecka A. Li B. Dhondt B. Hendrix A. Nieuwland R. (2022). Removal of platelets from blood plasma to improve the quality of extracellular vesicle research. J. Thromb. Haemost. 20 (11), 2679–2685. 10.1111/jth.15867 36043239 PMC9825910

[B6] Bettin B. A. Li B. Falkena K. van Leeuwen T. G. Gollwitzer C. Varga Z. (2025). Calibration of flow cytometers enables reproducible measurements of extracellular vesicle concentrations and reference range establishment. J. Extracell. Vesicles 14 (12), e70189. 10.1002/jev2.70189 41392534 PMC12703049

[B7] Botha J. Pugsley H. R. Handberg A. (2021). Conventional, High-Resolution and Imaging Flow Cytometry: Benchmarking Performance in Characterisation of Extracellular Vesicles. Biomedicines 9, 124. 10.3390/biomedicines9020124 33513846 PMC7911094

[B8] Brambilla M. Becchetti A. Nallio K. Camera M. (2024). Flow cytometry analysis of tissue factor expression in human platelets. JoVE 213, e67356. 10.3791/67356 39651739

[B9] Brihi J. E. Pathak S. (2024). Normal and Abnormal Complete Blood Count with Differential. Treasure Island, FL: StatPearls Publishing. Available online at: https://www.ncbi.nlm.nih.gov/books/NBK604207/ (Accessed January 10, 2026). 38861622

[B10] Chen C. Zong S. Wang Z. Lu J. Zhu D. Zhang Y. (2016). Imaging and intracellular tracking of cancer-derived exosomes using single-molecule localization-based super-resolution microscope. ACS Appl. Mater Interfaces 8 (39), 25825–25833. 10.1021/acsami.6b09442 27617891

[B11] Choi D. S. Kim D. K. Kim Y. K. Gho Y. S. (2015). Proteomics of extracellular vesicles: exosomes and ectosomes. Mass Spectrom. Rev. 34 (4), 474–490. 10.1002/mas.21420 24421117

[B12] Clayton A. Court J. Navabi H. Adams M. Mason M. D. Hobot J. A. (2001). Analysis of antigen presenting cell derived exosomes, based on immuno-magnetic isolation and flow cytometry. J. Immunol. Methods 247 (1), 163–174. 10.1016/s0022-1759(00)00321-5 11150547

[B13] Cohn J. S. McNamara J. R. Schaefer E. J. (1988). Lipoprotein cholesterol concentrations in the plasma of human subjects as measured in the fed and fasted states. Clin. Chem. 34 (12), 2456–2459. 3197284

[B14] Daaboul G. G. Gagni P. Benussi L. Bettotti P. Ciani M. Cretich M. (2016). Digital detection of exosomes by interferometric imaging. Sci. Rep. 6 (1), 37246. Available from. 10.1038/srep37246 27853258 PMC5112555

[B15] Dahmane S. Doucet C. Le Gall A. Chamontin C. Dosset P. Murcy F. (2019). Nanoscale organization of tetraspanins during HIV-1 budding by correlative dSTORM/AFM. Nanoscale 11 (13), 6036–6044. 10.1039/c8nr07269h 30869094

[B16] Dignat-George F. Boulanger C. M. (2011). The many faces of endothelial microparticles. Arterioscler. Thromb. Vasc. Biol. 31 (1), 27–33. 10.1161/ATVBAHA.110.218123 21160065

[B17] Février B. Raposo G. (2004). Exosomes: endosomal-derived vesicles shipping extracellular messages. Curr. Opin. Cell Biol. 16, 415–421. 10.1016/j.ceb.2004.06.003 15261674

[B18] Guduric-Fuchs J. O’Connor A. Camp B. O’Neill C. L. Medina R. J. Simpson D. A. (2012). Selective extracellular vesicle-mediated export of an overlapping set of microRNAs from multiple cell types. BMC Genomics 13, 357. 10.1186/1471-2164-13-357 22849433 PMC3532190

[B19] Han C. Kang H. Yi J. Kang M. Lee H. Kwon Y. (2021). Single-vesicle imaging and co-localization analysis for tetraspanin profiling of individual extracellular vesicles. J. Extracell. Vesicles 10 (3), e12047. 10.1002/jev2.12047 33456726 PMC7797949

[B20] Han C. Kang M. Kang H. Yi J. Lim M. Kwon Y. (2023). Characterization of extracellular vesicle and virus-like particles by single vesicle tetraspanin analysis. Sensors Actuators B Chem. 382, 133547. 10.1016/j.snb.2023.133547

[B21] Hessvik N. P. Sandvig K. Llorente A. (2013). Exosomal miRNAs as biomarkers for prostate cancer. Front. Genet. 4, 36. 10.3389/fgene.2013.00036 23519132 PMC3604630

[B22] Holcar M. Marić I. Tertel T. Goričar K. Čegovnik Primožič U. Černe D. (2025). Comprehensive phenotyping of extracellular vesicles in plasma of healthy humans - insights into cellular origin and biological variation. J. Extracell. Vesicles 14 (1), e70039. 10.1002/jev2.70039 39834131 PMC11746918

[B23] Im H. Shao H. Park Y. Peterson V. M. Castro C. M. Weissleder R. (2014). Label-free detection and molecular profiling of exosomes with a nano-plasmonic sensor. Nat. Biotechnol. 32 (5), 490–495. 10.1038/nbt.2886 24752081 PMC4356947

[B24] Ivanov I. I. Apta B. H. R. Bonna A. M. Harper M. T. (2019). Platelet P-selectin triggers rapid surface exposure of tissue factor in monocytes. Sci. Rep. 9 (1), 13397. 10.1038/s41598-019-49635-7 31527604 PMC6746844

[B25] Janowska-Wieczorek A. Marquez-Curtis L. A. Wysoczynski M. Ratajczak M. Z. (2006). Enhancing effect of platelet-derived microvesicles on the invasive potential of breast cancer cells. Transfusion 46 (7), 1199–1209. 10.1111/j.1537-2995.2006.00871.x 16836568

[B26] Jeong H. Han C. Cho S. Gianchandani Y. Park J. (2018). Analysis of extracellular vesicles using coffee ring. ACS Appl. Mater Interfaces 10 (27), 22877–22882. 10.1021/acsami.8b05793 29911857

[B27] Ji H. Chen M. Greening D. W. He W. Rai A. Zhang W. (2014). Deep sequencing of RNA from three different extracellular vesicle (EV) subtypes released from the human LIM1863 Colon cancer cell line uncovers distinct mirna-enrichment signatures. PLoS One 9 (10), e110314. 10.1371/journal.pone.0110314 25330373 PMC4201526

[B28] Johnsen K. B. Gudbergsson J. M. Andresen T. L. Simonsen J. B. (2019). What is the blood concentration of extracellular vesicles? Implications for the use of extracellular vesicles as blood-borne biomarkers of cancer. Biochim. Biophys. Acta Rev. Cancer 1871 (1), 109–116. 10.1016/j.bbcan.2018.11.006 30528756

[B29] Joop K. Berckmans R. J. Nieuwland R. Berkhout J. Romijn F. P. Hack C. E. (2001). Microparticles from patients with multiple organ dysfunction syndrome and sepsis support coagulation through multiple mechanisms. Thromb. Haemost. 85 (5), 810–820. 11372673

[B30] Jørgensen M. Bæk R. Pedersen S. Søndergaard EKLEKL Kristensen S. R. Varming K. (2013). Extracellular vesicle (EV) array: microarray capturing of exosomes and other extracellular vesicles for multiplexed phenotyping. J. Extracell. Vesicles 2 (1), 20920. 10.3402/jev.v2i0.20920 24009888 PMC3760630

[B31] Jørgensen M. M. M. Bæk R. Varming K. (2015). Potentials and capabilities of the extracellular vesicle (EV) array. J. Extracell. Vesicles 4 (1), 26048. 10.3402/jev.v4.26048 25862471 PMC4393420

[B32] Kao C. Y. Papoutsakis E. T. (2019). Extracellular vesicles: exosomes, microparticles, their parts, and their targets to enable their biomanufacturing and clinical applications. Curr. Opin. Biotechnol. 60, 89–98. 10.1016/j.copbio.2019.01.005 30851486

[B33] Keller Cecconello D. Spagnol F. Alegretti A. P. Pilger D. A. Farias M. G. (2024). Flow cytometry immunophenotyping of healthy platelets and hospitalized patients with suspected platelet dysfunction: challenges for establishing a cutoff value. Hematol. Transfus. Cell Ther. 46, S136–S142. 10.1016/j.htct.2023.07.002 37652805 PMC11670585

[B34] Knijff-Dutmer E. A. J. Koerts J. Nieuwland R. Kalsbeek-Batenburg E. M. van de Laar MAFJ (2002). Elevated levels of platelet microparticles are associated with disease activity in rheumatoid arthritis. Arthritis Rheum. 46 (6), 1498–1503. 10.1002/art.10312 12115179

[B35] Krämer-Albers E. M. Hill A. F. (2016). Extracellular vesicles: interneural shuttles of complex messages. Curr. Opin. Neurobiol. 39, 101–107. 10.1016/j.conb.2016.04.016 27183381

[B36] Krzyslak H. Szejniuk W. M. Falkmer U. Honoré B. Jørgensen M. M. Sten C. (2026). Methodological and short-term diurnal variation in surface and cargo proteins in plasma extracellular vesicles. Curr. Issues Mol. Biol. 48, 120. 10.3390/cimb48010120 41614950 PMC12840092

[B37] Kumar M. A. Baba S. K. Sadida H. Q. Marzooqi S. Al Jerobin J. Altemani F. H. (2024). Extracellular vesicles as tools and targets in therapy for diseases. Signal Transduct. Target Ther. 9 (1), 27. 10.1038/s41392-024-01735-1 38311623 PMC10838959

[B38] Lee W. Nanou A. Rikkert L. Coumans F. A. W. Otto C. Terstappen LWMM (2018). Label-free prostate cancer detection by characterization of extracellular vesicles using raman spectroscopy. Anal. Chem. 90 (19), 11290–11296. 10.1021/acs.analchem.8b01831 30157378 PMC6170952

[B39] Lee. K. Fraser K. Ghaddar B. Yang K. Kim E. Balaj L. (2018). Multiplexed profiling of single extracellular vesicles. ACS Nano 12 (1), 494–503. 10.1021/acsnano.7b07060 29286635 PMC5898240

[B40] Margolis L. Sadovsky Y. (2019). The biology of extracellular vesicles: the known unknowns. PLoS Biol. 17 (7), e3000363. 10.1371/journal.pbio.3000363 31318874 PMC6667152

[B41] Nielsen C. T. Østergaard O. Stener L. Iversen L. V. Truedsson L. Gullstrand B. (2012). Increased IgG on cell-derived plasma microparticles in systemic lupus erythematosus is associated with autoantibodies and complement activation. Arthritis Rheum. 64 (4), 1227–1236. 10.1002/art.34381 22238051

[B42] Nizamudeen Z. Markus R. Lodge R. Parmenter C. Platt M. Chakrabarti L. (2018). Rapid and accurate analysis of stem cell-derived extracellular vesicles with super resolution microscopy and live imaging. Biochim. Biophys. Acta Mol. Cell Res. 1865 (12), 1891–1900. 10.1016/j.bbamcr.2018.09.008 30290236 PMC6203808

[B43] Nomura S. Uehata S. Saito S. Osumi K. Ozeki Y. Kimura Y. (2003). Enzyme immunoassay detection of platelet-derived microparticles and RANTES in acute coronary syndrome. Thromb. Haemost. 89 (3), 506–512. 12624635

[B44] Østergaard O. Nielsen C. T. Iversen L. V. Tanassi J. T. Knudsen S. Jacobsen S. (2013). Unique protein signature of circulating microparticles in systemic lupus erythematosus. Arthritis Rheum. 65 (10), 2680–2690. 10.1002/art.38065 23817959

[B45] Raposo G. Stoorvogel W. (2013). Extracellular vesicles: exosomes, microvesicles, and friends. J. Cell Biol. 200 (4), 373–383. 10.1083/jcb.201211138 23420871 PMC3575529

[B46] Rasmussen R. W. Botha J. Prip F. Sanden M. Nielsen M. H. Handberg A. (2021). Zoom in on antibody aggregates: a potential pitfall in the search of rare EV populations. Biomedicines 9 (2), 206. 10.3390/biomedicines9020206 33670624 PMC7923005

[B47] Sheremata W. A. Jy W. Horstman L. L. Ahn Y. S. Alexander J. S. Minagar A. (2008). Evidence of platelet activation in multiple sclerosis. J. Neuroinflammation 5, 27. 10.1186/1742-2094-5-27 18588683 PMC2474601

[B48] Simpson R. J. Jensen S. S. Lim J. W. E. (2008). Proteomic profiling of exosomes: current perspectives. Proteomics 8 (19), 4083–4099. 10.1002/pmic.200800109 18780348

[B49] Skotland T. Hessvik N. P. Sandvig K. Llorente A. (2019). Exosomal lipid composition and the role of ether lipids and phosphoinositides in exosome biology. J. Lipid Res. 60 (1), 9–18. 10.1194/jlr.R084343 30076207 PMC6314266

[B50] Sódar B. W. Kittel Á. Pálóczi K. Vukman K. V. Osteikoetxea X. Szabó-Taylor K. (2016). Low-density lipoprotein mimics blood plasma-derived exosomes and microvesicles during isolation and detection. Sci. Rep. 6 (1), 24316. 10.1038/srep24316 27087061 PMC4834552

[B51] Théry C. Zitvogel L. Amigorena S. (2002). Exosomes: composition, biogenesis and function. Nat. Rev. Immunol. 2 (8), 569–579. 10.1038/nri855 12154376

[B52] Welsh J. A. Van Der Pol E. Arkesteijn G. J. A. Bremer M. Brisson A. Coumans F. (2020). MIFlowCyt-EV: a framework for standardized reporting of extracellular vesicle flow cytometry experiments. J. Extracell. Vesicles 9 (1), 1713526. 10.1080/20013078.2020.1713526 32128070 PMC7034442

[B53] Welsh J. A. Arkesteijn G. J. A. Bremer M. Cimorelli M. Dignat-George F. Giebel B. (2023). A compendium of single extracellular vesicle flow cytometry. J. Extracell. Vesicles 12 (2), e12299. 10.1002/jev2.12299 36759917 PMC9911638

[B54] Welsh J. A. Goberdhan D. C. I. O’Driscoll L. Buzas E. I. Blenkiron C. Bussolati B. (2024). Minimal information for studies of extracellular vesicles (MISEV2023): from basic to advanced approaches. J. Extracell. Vesicles 13 (2), e12404. 10.1002/jev2.12404 38326288 PMC10850029

[B55] Widjaja A. Morris R. J. Levy J. C. Frayn K. N. Manley S. E. Turner R. C. (1999). Within- and between-subject variation in commonly measured anthropometric and biochemical variables. Clin. Chem. 45 (4), 561–566. 10102917

[B56] Witwer K. W. Buzás E. I. Bemis L. T. Bora A. Lässer C. Lötvall J. (2013). Standardization of sample collection, isolation and analysis methods in extracellular vesicle research. J. Extracell. Vesicles 2. 10.3402/jev.v2i0.20360 24009894 PMC3760646

[B57] Yim K. H. W. Krzyzaniak O. Al Hrout A. Peacock B. Chahwan R. (2023). Assessing extracellular vesicles in human biofluids using flow-based analyzers. Adv. Healthc. Mater 12 (32), 2301706. 10.1002/adhm.202301706 37800440 PMC11469288

[B58] Zwicker J. I. Trenor C. C. 3rd Furie B. C. Furie B. (2011). Tissue factor-bearing microparticles and thrombus formation. Arterioscler. Thromb. Vasc. Biol. 31 (4), 728–733. 10.1161/ATVBAHA.109.200964 21252066 PMC6112768

