# Supplementary material for: Longitudinal changes in the phenotypic profile of circulating extracellular vesicles in healthy individuals
Source: Front Cell Dev Biol. 2026 Jun 19;14:1807969. doi: 10.3389/fcell.2026.1807969 (PMC13328366; doi:10.3389/fcell.2026.1807969)
Supplement: Supplementary file 1 [file Supplementaryfile1.docx]

Supplementary Material

**Supplementary Table S1** MIFlowCyt compliant item check list.

| **Requirement** | **Please Include Requested Information** |
| --- | --- |
| 1.1. Purpose | Investigate circulating levels of CD9^+^-EVs, CD81^+^-EVs, CD63^+^-EVs, CD9^+^CD81^+^-EVs, CD9^+^CD63^+^-EVs, and CD81^+^CD63^+^-EVs in circulation in healthy donors. |
| 1.2. Keywords | Extracellular vesicles; High resolution flow cytometry; Healthy donors; Variation |
| 1.3. Experiment variables | For all procedures, the same detailed protocols were used, and all steps were performed by the same two skilled laboratory technicians.   - Sample collection and preparation of platelet poor plasma (PPP) was done by skilled laboratory technicians and researchers, respectively. Details regarding these procedures are described in section 2.3. - Staining and hFCM analysis of PPPs. Details are described in Table S2, section 2.1, 2.3, and 4.1. - Analysis and gating in FlowJo were performed by skilled researchers. Details are described in section 4.4.1 and 4.4.3. |
| 1.4. Organization name and address | Department of Clinical Immunology, Aalborg University Hospital, North Denmark Region, Urbansgade 32-36, DK-9000 Aalborg, Denmark.  Department of Clinical Biochemistry, Aalborg University Hospital, North Denmark Region, Hobrovej 18-22, DK-9000 Aalborg, Denmark.  Department of Clinical Medicine, Aalborg University, Selma Lagerløfs Vej 249, DK-9020 Gistrup, Denmark. |
| 1.5. Primary contact name and email address | Maiken Mellergaard, maiken.pedersen@rn.dk |
| 1.6. Date or time period of experiment | 2023.08.23-2023.09.14 |
| 1.7. Conclusions | - Intra- and inter-individual variation in EV/particle numbers were more pronounced for long-term (week-to-week) compared with short-term (day-to-day) observations - Especially, CD9^+^- and to a lesser extent CD81^+^-EV assessment revealed lower CVs overall, across the different methods |
| 1.8. Quality control measures | **Titrations:**  Single isotype/antibody titration  Antibody panel titration  Sample serial dilution  **Daily system stability controls:**  Light scatter/volumetric control: Apogee Mix bead mixture (Apogee Flow Systems, Cat. no. 1527, Lot. no. CAL0172).  Fluorescence: Ultra Rainbow Calibration particles (Spherotech, Lake Forest, IL, USA, Cat. no. RCP-30-5, Lot. no. AP04).  Background stability: Buffer alone control (PBS, Merck, D8537, sterile filtered and suitable for cell culture).  **Sample-specific controls:**  Unstained buffer (PBS)  PBS + isotype mix  PBS + antibody mix  Unstained samples  Stained samples – isotype mix  Stained samples – antibody mix  Detergent lysis for Stained samples – antibody mix |
| 2.1.1.1. (2.1.2.1., 2.1.3.1.) Sample description | Platelet poor plasma (PPP). |
| 2.1.1.2. Biological sample source description | Blood samples were collected fasting. PPP (venous blood from which red blood cells, white blood cells, and (most) platelets have been removed), procedure described in section 2.3. |
| 2.1.1.3. Biological sample source organism description | Human |
| 2.1.2.2. Environmental sample location |  |
| 2.3. Sample treatment description | Venous blood was collected using a 21-gauge needle, and the first 3.5 mL of blood was discarded. The blood was collected through venous draw into EDTA K3 (4 mL), or CPDA (6 mL) Vacuette™ tubes (Greiner Bio-one GmbH, Austria, Cat. 456057). One tube of CPDA was centrifuged two times at 2,500 x g for 15 minutes (min) at room temperature (RT), plasma was isolated and aliquoted prior to storage at -40°C and subsequent hFCM analysis. |
| 2.4. Fluorescence reagent(s) description | **Antibodies and label:**  FITC-conjugated mouse monoclonal anti-CD9 (Clone HI9a; Biolegend; Cat. 312104, Lot: B359149), PE-conjugated mouse monoclonal anti-CD81 (Clone 5A6; Biolegend; Cat. 349506, Lot: B346939), APC-conjugated mouse monoclonal anti-CD63 (Clone H5C6; Biolegend; Cat. 353008; Lot: B373947).  **Isotype controls:**  FITC-conjugated mouse monoclonal IgG1κ isotype control (Clone MOPC-21; Biolegend; Cat. 400108; Lot: B258679), PE-conjugated mouse monoclonal IgG1κ isotype control (Clone MOPC-21; Biolegend; Cat. 400114; Lot: B245983), and APC-conjugated mouse monoclonal IgG1κ isotype control (Clone MOPC-21; Biolegend; Cat. 400122; Lot: B271888). |
| 3.1. Instrument manufacturer | Apogee Flow Systems, Hemel Hempstead, UK |
| 3.2. Instrument model | A60 Micro-PLUS |
| 3.3. Instrument configuration and settings | Instrument configuration and settings are shown in Table S3. |
| 4.1. List-mode data files | Email to: Maiken Mellergaard, maiken.pedersen@rn.dk |
| 4.2. Compensation description | Compensation was performed using compensation beads (Spherotech, Cat. CMIg-08-2K, Lot: AM01) for FITC-CD9 (2.5 µL) and PE-CD81 (5 µL), and APC-CD63 (5 µL). Unstained samples and FMO controls were included to define fluorescence spill-over. Same antibody concentrations as for samples were used. Same flow cytometry settings (Table S3). Compensation matrix is shown in Figure S3. |
| 4.3. Data transformation details | Not applicable. |
| 4.4.1. Gate description | Gates were set according to isotype controls. Positive events were defined as having a fluorescent signal higher than the lower boundary of the gate (see Figure S4 for gating strategy). |
| 4.4.2. Gate statistics | Concentrations were calculated as number of events divided by sample volume and multiplied by dilution factor. |
| 4.4.3. Gate boundaries | Gate boundaries were defined according to Rosetta beads for size and isotype controls for background (see Figure S4 for gating strategy). |

**Supplementary Table S2** MIFlowCyt-EV compliant item checklist.

| **Framework Criteria** | **What to report** | **Please complete each criterion** |
| --- | --- | --- |
| 1.1 Preanalytical variables conforming to MISEV guidelines. | Preanalytical variables relating to EV sample including source, collection, isolation, storage, and any others relevant and available in the performed study. | Described in Table S1, section 2.1.1.2 and 2.3 |
| 1.2 Experimental design according to MIFlowCyt guidelines. | EV-FC manuscripts should provide a brief description of the experimental aim, keywords, and variables for the performed FC experiment(s) using MIFlowCyt checklist criteria: 1.1, 1.2, and 1.3, respectively | Described in Table S1, section 1.1, 1.2, and 1.3. |
| 2.1 Sample staining details | State any steps relating to the staining of samples. Along with the method used for staining, provide relevant reagent descriptions as listed in MIFlowCyt guidelines (Section 2.4 Fluorescence Reagent(s) Descriptions). | Details about antibodies, label, and isotypes are descried in Table S1, section 2.4.  **Titrations and antibody/isotype mixes:**  Titration of all antibodies, label, and isotypes were performed on PPP to define optimal concentration ensuring optimal labelling without excess to enhance risk of unspecific binding and antibody aggregation. Final concentrations were used for mixes as described:  Antibody mixes were prepared by adding FITC-CD9 (2.5 µL/sample), PE-C81 (5 µL/sample), and APC-CD63 (5 µL/sample) in 37.5 µL PBS/sample.  Isotype control mixes (background controls, added in same concentrations as specific antibodies) were prepared by adding FITC- IgG1κ isotype control (0.5 µL/sample), PE- IgG1κ isotype control (3 µL/sample), and APC-IgG1κ isotype control (5 µL/sample) in 41.5 µL PBS/sample.  **Staining of samples:**  The thawed PPP sample was subjected to a centrifugation cycle at 1850xg for 5 min at 22°C after which supernatant (leaving approximately 5-10mm plasma above pellet) was collected to remove debris formed during freezing and thawing.  Preparation of antibody and isotype mix solutions: Prior to staining antibody and isotype mixes were prepared in PBS and filtered through 0,45 µm centrifugation filters (Millipore, Cat. UCF30HVNB) at 12000xg for 10 min at 22°C to reduce antibody aggregates.  Staining was performed by mixing 10 µL of PPP sample and 50 µL antibody or isotype mixes or PBS (for unstained control). Samples were mixed thoroughly by vortexing and incubated at RT in the dark for 2 h.  After incubation, samples were diluted individually in PBS according to dilution factor (described in section 2.3 below) and kept dark at RT until analysis. |
| 2.2 Sample washing details | State any steps relating to the washing of samples. | No sample washing was performed as all PPP samples were stained directly in PBS-antibody mixes and only further diluted in PBS prior to flow cytometry analysis. |
| 2.3 Sample dilution details | All methods and steps relating to sample dilution. | All samples were diluted 1:6 for staining (in antibody mix, isotype mix, or PBS; 10 µL of PPP sample was diluted in 50 µL antibody or isotype mixes or PBS).  After incubation, samples were diluted individually in PBS according to dilution factor determined by titrating unstained sample (dilution factor between 1:50 and 1:800) to obtain an optimal flowrate (described in section 4.1). |
| 3.1 Buffer alone controls. | State whether a buffer-only control was analyzed at the same settings and during the same experiment as the samples of interest. If utilized it is recommended that all samples be recorded for a consistent set period of time e.g. 5 minutes, rather than stopping analysis at a set recorded event count e.g. 100,000 events. This allows comparisons of total particle counts between controls and samples. | Buffer alone (PBS) controls were included every day at start up as well as between each sample after cleaning solution to monitor and ensure stable (and low, defined as < 100 events/sec) background of the instrument. Clean and PBS between samples were analyzed with settings: 10.5 μL/min, aspirating 200 µL, acquisition time set to 180 sec. |
| 3.2 Buffer with reagent controls. | State whether a buffer with reagent control was analyzed at the same settings, same concentrations, and during the same experiment as the samples of interest. If used state what the results were. | Buffer (PBS) with antibody mixes or isotype mixes (antibody aggregate controls) were included every week, analyzed according to the same settings as the samples (settings: 3.01 μL/min, aspirating 120 µL, acquisition time set to 180 sec.) to access and define potential background.  Since some antibody aggregates were observed when accessing CD81^+^ events as single positive events (488-orange vs MALS), these events were gated out to not interfere with the final EV concentrations, as shown in Figure S4. |
| 3.3 Unstained controls. | State whether unstained control samples were analyzed at the same settings and during the same experiment as stained samples. If used, state what the results were, preferably in standard units. | Unstained sample controls were included for each sample to access and define background autofluorescence. Unstained controls were analyzed according to the same settings as the samples (settings: 3.01 μL/min, aspirating 120 µL, acquisition time set to 180 sec.). |
| 3.4 Isotype controls. | The use of isotype controls is applicable to immunofluorescence labelling only. State whether isotype controls were analyzed at the same settings and during the same experiment as stained samples. If utilized, state which antibody they are matched to, the concentration used, and what the results were (Section 4.2, 4.3, 4.4). Due to conjugation differences between manufacturers if should be stated if the isotype controls are from the same manufacturer as the matched antibodies. | Isotype controls were included for each sample to access and define background and possible unspecific antibody binding. Isotype controls were analyzed according to the same settings as the samples (settings: 3.01 μL/min, aspirating 120 µL, acquisition time set to 180 sec.). Isotype controls were purchased from the same manufacturer as the matched antibodies (see details in Table S1 section 2.4, Table S2 2.1, and Figure S4). |
| 3.5 Single-stained controls. | State whether single-stained controls were included. If used state whether the single-stained controls were recorded using the same settings, dilutions, and during the same experiment as stained samples and state what the results were, preferably in standard units (Section 4.2, 4.3, 4.4). | Single stained controls were included in the study preparation and titration of antibodies (described in section 2.1), but not for each sample throughout the study. |
| 3.6 Procedural controls. | State whether procedural controls were included. If used, state the procedure and if the procedural controls were acquired at the same settings and during the same experiment as stained samples. | No procedural controls were included. |
| 3.7 Serial dilutions. | State whether serial dilutions were performed on samples and note the dilution range and manner of testing. The fluorescence and/or scatter signal intensity would ideally be reported in standard units (see Section 4.3, 4.4) but arbitrary units can also be used. This data is best reported by plotting the recorded number events/concentration over a set period of time at different sample dilution. The median fluorescence intensity at each of the dilutions should also ideally be plotted on the same or a separate plot. | Serial dilutions of stained (antibody mix) samples (analysis of serial dilutions: 1:100, 1:200, 1:300, 1:400, 1:500) of stained samples were performed to define and secure the optimal dilution of stained samples and avoid swarm detection. Optimal flowrate, described in 4.1. |
| 3.8. Detergent treated EV-samples | State whether samples were detergent treated to assess lability. If utilized, state what detergent was used, the end concentration of the detergent, and what the results were of the lysis. | Detergent lysis controls were included for all (antibody mix) stained samples incubating stained samples in a 1 % (final concentration) Triton X-100 (Merck, Cat. 93443-100mL) for at least 30 minutes at RT in the dark. Detergent lysis controls were analyzed according to the same settings as the samples (settings: 3.01 μL/min, aspirating 120 µL, acquisition time set to 180 sec.), exemplified in Figure S4. |
| 4.1 Trigger Channel(s) and Threshold(s). | The trigger channel(s) and threshold(s) used for event detection. Preferably, the fluorescence calibration (Section 4.3) and/or scatter calibration (Section 4.4) should be used in order to report the trigger channel(s) and threshold(s) in standardized units. | A triggering threshold was set on medium-angle light scatter (MALS) to a value of 27 (= 1728 units in MALS, FCS files). This setting allowed the collection of less than 100 events/second in unstained PBS. Optimal flowrate of 4000-6000 events/sec in unlabelled PPP samples were titrated individually for each sample. |
| 4.2 Flow Rate / Volumetric quantification. | State if the flow rate was quantified/validated and if so, report the result and how they were obtained. | Apogee Mix bead mixture (Apogee Flow Systems, Cat. no. 1527, Lot. no. CAL0158) were run daily to validate Apogee instrument performance.  Concentration of 110 nm polystyrene beads were collected and plotted against known concentration of 110 nm beads. Moreover, proper separation between all nine bead populations were monitored. |
| 4.3 Fluorescence Calibration. | State whether fluorescence calibration was implemented, and if so, report the materials and methods used, catalogue numbers, lot numbers, and supplied reference units for the standards. Fluorescence parameters may be reported in standardized units of MESF, ERF, or ABC beads. The type of regression used, and the resulting scatter plot of arbitrary data vs standard data for the reference particles should be supplied. | FITC, PE, and APC fluorescence were calibrated into standardized Molecules of Equivalent Soluble Fluorochrome (MESF) units using fluorescent beads (Spherotech beads: Cat. ECFP-F1-5K, Lot: AQ01; Cat. ECFP-F2-5K, Lot: AN01; Cat. ACP-30-5K, Lot: AN01; Cat. RCP-30-5, Lot: AP04). Log10-transformed MFI and log10-transformed MESF were used for regression analysis (shown in Figure S2). |
| 4.4 Light Scatter Calibration. | State whether and how light scatter calibration was implemented. Light scatter parameters may be reported in standardized units of nm2, along with information required to reproduce the model. | Rosetta beads, Exometry, Cat. CAL003, Lot: KF002 were used to calibrate and convert arbitrary side scatter units to size (nm) according to manufacturer’s guidelines (FCM settings and sample calculation parameters are shown in figure S1). |
| 5.1 EV diameter/surface area/volume approximation. | State whether and how EV diameter, surface area, and/or volume has been calculated using FC measurements. | Rosetta beads, Exometry, Cat. CAL003, Lot: KF002 were used to calibrate and convert arbitrary side scatter units to size (nm) according to manufacturer’s guidelines. Size was utilized for gating on 0-200, 110-1000, and 0-1000 nm particles, as shown in Figure S4. |
| 5.2 EV refractive index approximation. | State whether the EV refractive index has been approximated and how this was done. | EV refractive index was not approximated in this study. |
| 5.3 EV epitope number approximation. | State whether EV epitope number has been approximated, and if so, how it was approximated. | Not relevant. |
| 6.1 Completion of MIFlowCyt checklist. | Complete MIFlowCyt checklist criteria 1 to 4 using the MIFlowCyt guidelines. | MIFlowCyt checklist has been completed and is provided separately (Table S1). |
| 6.2 Calibrated channel detection range | If fluorescence or scatter calibration has been carried out, authors should state whether the upper and lower limits of a calibrated detection channel were calculated in standardized units. This can be done by converting the arbitrary unit scale to a calibrated scaled, as discussed in Section 4.3 and 4.4, and providing the highest unit on this scale and the lowest detectable unit above the unstained population. The lowest unit at which a population is deemed ‘positive’ can be determined a variety of ways, including reporting the 99th percentile measurement unit of the unstained population for fluorescence. The chosen method for determining at what unit an event was deemed positive should be clearly outlined. | See Figure S2 and S4. |
| 6.3 EV number/concentration. | State whether EV number/concentration has been reported. If calculated, it is preferable to report EV number/concentration in a standardized manner, stating the number/concentration between a set detection range. | EV concentrations were calculated for the different EV-populations as EVs/mL (as described in Table S1, section 4.4.2). |
| 6.4 EV brightness. | When applicable, state the method by which the brightness of EVs is reported in standardized units of scatter and/or fluorescence. |  |
| 7.1. Sharing of data to a public repository. | Provide a link to the experimental data in a public data repository. | Contact Maiken Mellergaard, maiken.pedersen@rn.dk |

**Supplementary Table S3** Flow cytometer settings.

| **Marker** | **Fluorophore** | **Channel settings** | **Threshold** | **Notes** |
| --- | --- | --- | --- | --- |
| **SALS** | - | Laser: 405nm (300nW)  Setting: 140mW  Filter: 445/50  PMT: 400V | - |  |
| **MALS** | - | Laser: 405nm (300mW)  Setting: 140mW  Filter: 525/50  PMT: 380V | 27 | Background in PBS: < 100 events/s |
| **LALS** | - | Laser: 405nm (300mW)  Setting: 140mW  Filter: LP575  PMT: 400V | - |  |
| **CD9** | FITC | Laser: 488nm (200 mW)  Setting: 100mW  Filter: 525/50 PMT: 400V | - |  |
| **CD81** | PE | Laser: 488nm (200 mW)  Setting: 100mW  Filter: 575/30 PMT: 400V | - |  |
| **CD63** | APC | Laser: 638nm (180 mW)  Setting: 100mW  Filter: 680/35 PMT: 400V | - |  |

**Supplementary Table S4** Short- and long-term blood counts measured with SYSMEX for four donors (A-D) of erythrocytes (ERYT), platelets (PLT), leukocytes (LEUK), lymphocytes (LYMPH), monocytes (MONO), neutrophils (NEUT), eosinophils (EO) and basophils (BASO).

| **LEUK (×10^9^ cells/L)** | | | | | | | | | | |
| --- | --- | --- | --- | --- | --- | --- | --- | --- | --- | --- |
| Day | A | B | C | D | Week | A | B | C | D | Normal range |
| 1 | 7.0 | 7.2 | 5.2 | 4.1 | 1 | 9.0 | 4.9 | 6.4 | 4.6 | 3.5-11.0 |
| 2 | 5.1 | 6.3 | 6.6 | 5.1 | 2 | 7.4 | 5.7 | 6.3 | 4.7 |  |
| 3 | 5.3 | 5.7 | 7.0 | 5.5 | 3 | 7.0 | 7.2 | 5.2 | 4.1 |  |
| 4 | 5.5 | 7.0 | 5.7 | 5.4 | 4 | 6.9 | 5.8 | 5.7 | 4.5 |  |
| 5 | 5.9 | 5.5 | 5.7 | 4.3 | 5 | 6.6 | 5.7 | **11.1** | 5.1 |  |
|  |  |  |  |  | 6 | 8.6 | 5.4 | 6.2 | 6.3 |  |
| Min | 5.1 | 5.5 | 5.2 | 4.1 | Min | 6.6 | 4.9 | 5.2 | 4.1 |  |
| Max | 7.0 | 7.2 | 7.0 | 5.5 | Max | 9.0 | 7.2 | 11.1 | 6.3 |  |
| Diff | 1.9 | 1.7 | 1.9 | 1.4 | Diff | 2.4 | 2.3 | 5.9 | 2.2 |  |
| **ERYT (×10^12^ cells/L)** | | | | | | | | | | |
| Day | A | B | C | D | Week | A | B | C | D | Normal range |
| 1 | 4.6 | **3.7** | 4.6 | 4.7 | 1 | 4.7 | 4.1 | 4.2 | 4.6 | 4.2-5.4 |
| 2 | 4.4 | 4.2 | 4.5 | 4.9 | 2 | 4.4 | 4.0 | 4.7 | 4.9 |  |
| 3 | 4.4 | 4.1 | 4.4 | 4.7 | 3 | 4.6 | **3.7** | 4.6 | 4.7 |  |
| 4 | 4.4 | 4.2 | 4.5 | 4.7 | 4 | 4.4 | 4.0 | 4.6 | 4.9 |  |
| 5 | 4.5 | 4.3 | 4.3 | 4.6 | 5 | 4.8 | 4.3 | 4.5 | 4.7 |  |
|  |  |  |  |  | 6 | 4.8 | 4.0 | 4.4 | 4.8 |  |
| Min | 4.4 | 3.7 | 4.3 | 4.6 | Min | 4.4 | 3.7 | 4.2 | 4.6 |  |
| Max | 4.6 | 4.3 | 4.6 | 4.9 | Max | 4.8 | 4.3 | 4.7 | 4.9 |  |
| Diff | 0.2 | 0.7 | 0.2 | 0.3 | Diff | 0.4 | 0.6 | 0.4 | 0.3 |  |
| **PLT (×10^9^ cells/L)** | | | | | | | | | | |
| Day | A | B | C | D | Week | A | B | C | D | Normal range |
| 1 | 272 | 234 | 240 | 229 | 1 | 311 | 211 | 169 | 205 | 150-400 |
| 2 | 278 | 247 | 233 | 244 | 2 | 312 | 207 | 223 | 224 |  |
| 3 | 250 | 244 | 225 | 226 | 3 | 272 | 234 | 240 | 229 |  |
| 4 | 259 | 240 | 227 | 226 | 4 | 269 | 239 | 223 | 225 |  |
| 5 | 271 | 251 | 221 | 221 | 5 | 310 | 223 | 207 | 223 |  |
|  |  |  |  |  | 6 | 285 | 198 | 202 | 231 |  |
| Min | 250 | 234 | 221 | 221 | Min | 269 | 198 | 169 | 205 |  |
| Max | 278 | 251 | 240 | 244 | Max | 312 | 239 | 240 | 231 |  |
| Diff | 28 | 17 | 19 | 23 | Diff | 43 | 41 | 71 | 26 |  |
| **BASO (×10^9^ cells/L)** | | | | | | | | | | |
| Day | A | B | C | D | Week | A | B | C | D | Normal range |
| 1 | 0.05 | 0.04 | 0.02 | 0.01 | 1 | 0.04 | 0.05 | 0.02 | 0.02 | <0.20 |
| 2 | 0.03 | 0.03 | 0.01 | 0.01 | 2 | 0.06 | 0.03 | 0.03 | 0.01 |  |
| 3 | 0.04 | 0.04 | 0.02 | 0.02 | 3 | 0.05 | 0.04 | 0.02 | 0.01 |  |
| 4 | 0.03 | 0.06 | 0.02 | 0.02 | 4 | 0.04 | 0.04 | 0.03 | 0.01 |  |
| 5 | 0.04 | 0.06 | 0.02 | 0.02 | 5 | 0.05 | 0.04 | 0.04 | 0.02 |  |
|  |  |  |  |  | 6 | 0.04 | 0.05 | 0.02 | 0.02 |  |
| Min | 0.03 | 0.03 | 0.01 | 0.01 | Min | 0.04 | 0.03 | 0.02 | 0.01 |  |
| Max | 0.05 | 0.06 | 0.02 | 0.02 | Max | 0.06 | 0.05 | 0.04 | 0.02 |  |
| Diff | 0.02 | 0.03 | 0.01 | 0.01 | Diff | 0.02 | 0.02 | 0.02 | 0.01 |  |
| **EO (×10^9^ cells/L)** | | | | | | | | | | |
| Day | A | B | C | D | Week | A | B | C | D | Normal range |
| 1 | 0.4 | 0.2 | 0.3 | 0.1 | 1 | **0.5** | 0.1 | 0.4 | 0.0 | < 0.5 |
| 2 | 0.3 | 0.2 | **3.0** | 0.0 | 2 | **0.5** | 0.2 | 0.4 | 0.1 |  |
| 3 | 0.3 | 0.2 | 0.3 | 0.1 | 3 | 0.4 | 0.2 | 0.3 | 0.1 |  |
| 4 | 0.3 | 0.2 | 0.3 | 0.1 | 4 | **0.5** | 0.2 | 0.3 | 0.0 |  |
| 5 | 0.3 | 0.1 | 0.3 | 0.0 | 5 | **0.5** | 0.2 | 0.3 | 0.1 |  |
|  |  |  |  |  | 6 | 0.4 | 0.2 | 0.3 | 0.1 |  |
| Min | 0.3 | 0.1 | 0.3 | 0.0 | Min | 0.4 | 0.1 | 0.3 | 0.0 |  |
| Max | 0.4 | 0.2 | 3.0 | 0.1 | Max | 0.5 | 0.2 | 0.4 | 0.1 |  |
| Diff | 0.1 | 0.0 | 2.7 | 0.0 | Diff | 0.1 | 0.1 | 0.2 | 0.0 |  |
| **LYMPH (×10^9^ cells/L)** | | | | | | | | | | |
| Day | A | B | C | D | Week | A | B | C | D | Normal range |
| 1 | 2.2 | 1.5 | 1.5 | 1.7 | 1 | 2.1 | 1.5 | 1.6 | 1.8 | 1.0-4.0 |
| 2 | 1.7 | 1.7 | 1.8 | 1.9 | 2 | 1.9 | 1.3 | 1.8 | 1.8 |  |
| 3 | 1.8 | 1.8 | 1.9 | 1.7 | 3 | 2.2 | 1.5 | 1.5 | 1.7 |  |
| 4 | 1.9 | 1.6 | 1.7 | 1.9 | 4 | 2.0 | 1.9 | 1.6 | 1.8 |  |
| 5 | 1.9 | 1.5 | 1.9 | 1.6 | 5 | 2.0 | 1.4 | 1.8 | 1.9 |  |
|  |  |  |  |  | 6 | 2.2 | 1.6 | 1.7 | 2.4 |  |
| Min | 1.7 | 1.5 | 1.5 | 1.6 | Min | 1.9 | 1.3 | 1.5 | 1.7 |  |
| Max | 2.2 | 1.8 | 1.9 | 1.9 | Max | 2.2 | 1.9 | 1.8 | 2.4 |  |
| Diff | 0.4 | 0.3 | 0.4 | 0.2 | Diff | 0.3 | 0.6 | 0.3 | 0.7 |  |
| **MONO (×10^9^ cells/L)** | | | | | | | | | | |
| Day | A | B | C | D | Week | A | B | C | D | Normal range |
| 1 | 0.6 | 0.5 | 0.4 | 0.4 | 1 | 0.8 | 0.3 | 0.5 | 0.4 | 0.2-1.0 |
| 2 | 0.4 | 0.4 | 0.5 | 0.4 | 2 | 0.6 | 0.5 | 0.4 | 0.4 |  |
| 3 | 0.5 | 0.5 | 0.5 | 0.4 | 3 | 0.6 | 0.5 | 0.4 | 0.4 |  |
| 4 | 0.5 | 0.4 | 0.4 | 0.4 | 4 | 0.6 | 0.5 | 0.4 | 0.4 |  |
| 5 | 0.5 | 0.4 | 0.4 | 0.3 | 5 | 0.5 | 0.4 | 0.5 | 0.4 |  |
|  |  |  |  |  | 6 | 0.6 | 0.4 | 0.4 | 0.5 |  |
| Min | 0.4 | 0.4 | 0.4 | 0.3 | Min | 0.5 | 0.3 | 0.4 | 0.4 |  |
| Max | 0.6 | 0.5 | 0.5 | 0.4 | Max | 0.8 | 0.5 | 0.5 | 0.5 |  |
| Diff | 0.2 | 0.2 | 0.1 | 0.1 | Diff | 0.3 | 0.2 | 0.1 | 0.2 |  |
| **NEUT (×10^9^ cells/L)** | | | | | | | | | | |
| Day | A | B | C | D | Week | A | B | C | D | Normal range |
| 1 | 3.8 | 4.9 | 3.0 | 2.0 | 1 | 5.6 | 2.8 | 4.0 | 2.4 | 1.5-8.0 |
| 2 | 2.6 | 3.9 | 4.0 | 2.7 | 2 | 4.3 | 3.7 | 3.6 | 2.5 |  |
| 3 | 2.7 | 3.2 | 4.4 | 3.2 | 3 | 3.8 | 4.9 | 3.0 | 2.0 |  |
| 4 | 2.7 | 4.7 | 3.4 | 3.0 | 4 | 3.8 | 3.2 | 3.3 | 2.3 |  |
| 5 | 3.1 | 3.4 | 3.0 | 2.3 | 5 | 3.6 | 3.7 | **8.4** | 2.7 |  |
|  |  |  |  |  | 6 | 5.3 | 3.1 | 3.7 | 3.3 |  |
| Min | 2.6 | 3.2 | 3.0 | 2.0 | Min | 3.6 | 2.8 | 3.0 | 2.0 |  |
| Max | 3.8 | 4.9 | 4.4 | 3.2 | Max | 5.6 | 4.9 | 8.4 | 3.3 |  |
| Diff | 1.2 | 1.7 | 1.4 | 1.3 | Diff | 2.0 | 2.1 | 5.5 | 1.3 |  |

*Red shows datapoints outside the normal range*

**Supplementary Table S5** Short-and long-term nanoparticle tracking analysis (NTA) counts and sizes for four donors (A-D).

| **NTA (number) ×10^12^** | | | | | | | | | |
| --- | --- | --- | --- | --- | --- | --- | --- | --- | --- |
| Day | A | B | C | D | Week | A | B | C | D |
| 1 | 0.96 | 0.47 | 0.62 | 0.49 | 1 | 1.52 | 0.58 | 0.71 | 0.31 |
| 2 | 0.37 | 0.48 | 1.30 | 0.42 | 2 | 1.50 | 0.49 | 1.70 | 0.57 |
| 3 | 0.38 | 0.17 | 0.40 | 0.41 | 3 | 0.96 | 0.47 | 0.62 | 0.49 |
| 4 | 0.56 | 0.28 | 0.70 | 0.30 | 4 | 0.69 | 0.36 | 1.30 | 0.33 |
| 5 | 0.39 | 0.21 | 1.70 | 0.33 | 5 | 0.69 | 0.78 | 1.40 | 0.38 |
|  |  |  |  |  | 6 | 0.68 | 0.29 | 1.70 | 0.34 |
| Min | 0.37 | 0.17 | 0.40 | 0.30 | Min | 0.69 | 0.36 | 0.62 | 0.31 |
| Max | 0.96 | 0.48 | 1.70 | 0.49 | Max | 1.52 | 0.78 | 1.70 | 0.57 |
| Diff | 0.59 | 0.31 | 1.30 | 0.19 | Diff | 0.83 | 0.42 | 1.08 | 0.26 |
| **NTA (size) ×50** | | | | | | | | | |
| Day | A | B | C | D | Week | A | B | C | D |
| 1 | 108.6 | 111.1 | 101.2 | 95.2 | 1 | 108.8 | 98.3 | 101.3 | 111.8 |
| 2 | 109.4 | 97.4 | 112.8 | 104.8 | 2 | 118.5 | 96.9 | 98.1 | 97.0 |
| 3 | 96.8 | 91.4 | 101.9 | 96.5 | 3 | 108.6 | 111.1 | 101.2 | 95.2 |
| 4 | 100.7 | 93.2 | 108.1 | 104.1 | 4 | 103.0 | 89.8 | 110.2 | 100.4 |
| 5 | 103.5 | 95.8 | 103.6 | 102.4 | 5 | 101.6 | 97.2 | 114.3 | 102.9 |
|  |  |  |  |  | 6 | 111.8 | 90.1 | 107.4 | 99.5 |
| Min | 96.8 | 91.4 | 101.2 | 95.2 | Min | 101.6 | 89.8 | 98.1 | 95.2 |
| Max | 109.4 | 111.1 | 112.8 | 104.8 | Max | 118.5 | 111.1 | 114.3 | 111.8 |
| Diff | 12.6 | 19.7 | 11.6 | 9.6 | Diff | 16.9 | 21.3 | 16.2 | 16.6 |

**Supplementary Table S6** Short- and long-term high‑resolution flow cytometry (hFCM) cell counts of CD9+, CD63+ and CD81+ cells for four donors (A-D).

| **CD9 0-1000 nm (x10^7^ EVs/mL)** | | | | | | | | | |
| --- | --- | --- | --- | --- | --- | --- | --- | --- | --- |
| Day | A | B | C | D | Week | A | B | C | D |
| 1 | 17.8 | 17.3 | 31.7 | 44.0 | 1 | 31.3 | 30.0 | 30.2 | 63.5 |
| 2 | 28.9 | 41.7 | 26.2 | 31.8 | 2 | 35.0 | 39.7 | 57.0 | 48.0 |
| 3 | 33.5 | 14.3 | 16.6 | 23.7 | 3 | 17.8 | 17.3 | 31.7 | 44.0 |
| 4 | 43.9 | 21.1 | 19.7 | 21.0 | 4 | 21.0 | 27.0 | 52.5 | 22.0 |
| 5 | 31.0 | 13.6 | 17.7 | 18.6 | 5 | 48.2 | 30.9 | 36.6 | 29.3 |
|  |  |  |  |  | 6 | 35.8 | 56.8 | 31.5 | 41.4 |
| Min | 17.8 | 13.6 | 16.6 | 18.6 | Min | 17.8 | 17.3 | 30.2 | 22.0 |
| Max | 43.9 | 41.7 | 31.7 | 44.0 | Max | 48.2 | 56.8 | 57.0 | 63.5 |
| Diff | 26.0 | 28.0 | 15.0 | 25.4 | Diff | 30.4 | 39.5 | 26.8 | 41.5 |
| **CD63 0-1000 nm (x10^7^ EVs/mL)** | | | | | | | | | |
| Day | A | B | C | D | Week | A | B | C | D |
| 1 | 0.11 | 0.12 | 0.17 | 0.17 | 1 | 0.44 | 0.42 | 0.54 | 0.14 |
| 2 | 0.22 | 0.20 | 0.43 | 0.22 | 2 | 0.43 | 0.81 | 0.74 | 0.19 |
| 3 | 0.35 | 0.12 | 0.20 | 0.13 | 3 | 0.11 | 0.12 | 0.17 | 0.17 |
| 4 | 0.24 | 0.10 | 0.27 | 0.08 | 4 | 0.08 | 0.03 | 0.50 | 0.16 |
| 5 | 0.22 | 0.08 | 0.71 | 0.13 | 5 | 0.46 | 0.23 | 3.12 | 0.19 |
|  |  |  |  |  | 6 | 2.19 | 0.14 | 0.51 | 0.18 |
| Min | 0.11 | 0.08 | 0.17 | 0.08 | Min | 0.08 | 0.03 | 0.17 | 0.14 |
| Max | 0.35 | 0.20 | 0.71 | 0.22 | Max | 2.19 | 0.81 | 3.12 | 0.19 |
| Diff | 0.24 | 0.12 | 0.54 | 0.13 | Diff | 2.11 | 0.78 | 2.95 | 0.05 |
| **CD81 0-1000 nm (x10^7^ EVs/mL)** | | | | | | | | | |
| Day | A | B | C | D | Week | A | B | C | D |
| 1 | 0.90 | 1.00 | 1.10 | 1.11 | 1 | 2.12 | 1.70 | 3.95 | 1.29 |
| 2 | 1.20 | 1.78 | 3.37 | 1.58 | 2 | 2.30 | 1.52 | 4.70 | 1.82 |
| 3 | 1.84 | 0.61 | 0.94 | 1.67 | 3 | 0.90 | 1.00 | 1.10 | 1.11 |
| 4 | 1.91 | 1.19 | 1.72 | 0.97 | 4 | 1.13 | 0.42 | 5.20 | 1.39 |
| 5 | 1.36 | 1.50 | 5.91 | 2.23 | 5 | 1.49 | 0.78 | 10.53 | 1.56 |
|  |  |  |  |  | 6 | 2.31 | 1.76 | 8.01 | 1.25 |
| Min | 0.90 | 0.61 | 0.94 | 0.97 | Min | 0.90 | 0.42 | 1.10 | 1.11 |
| Max | 1.91 | 1.78 | 5.91 | 2.23 | Max | 2.31 | 1.76 | 10.53 | 1.82 |
| Diff | 1.01 | 1.17 | 4.97 | 1.26 | Diff | 1.40 | 1.34 | 9.43 | 0.71 |

**Supplementary Table S7** High‑resolution flow cytometry (hFCM) co-localization counts and coefficient of variance (CV) for four donors (A-D).

|  | **hFCM** | | | | | | | |
| --- | --- | --- | --- | --- | --- | --- | --- | --- |
|  | **D** | CD9∙CD81 | CD9∙CD63 | CD81∙CD63 | **W** | CD9∙CD81 | CD9∙CD63 | CD81∙CD63 |
| **Donor A** | **1** | 2,082,000 | 144,000 | 122,000 | **1** | 4,385,000 | 831,000 | 66,400 |
|  | **2** | 3,821,000 | 116,000 | 66,400 | **2** | 4,950,000 | 565,000 | 99,700 |
|  | **3** | 5,465,000 | 266,000 | 99,700 | **3** | 2,082,000 | 144,000 | 122,000 |
|  | **4** | 5,050,000 | 216,000 | 16,600 | **4** | 1,761,000 | 188,000 | 22,100 |
|  | **5** | 3,140,000 | 199,000 | 83,100 | **5** | 6,168,000 | 775,000 | 133,000 |
|  |  |  |  |  | **6** | 12,226,000 | 1,229,000 | 233,000 |
|  | **CV%** | 35.38 | 31.56 | 51.32 | **CV%** | 72.39 | 66.51 | 63.36 |
| **Donor B** | **1** | 1,268,000 | 94,100 | 44,300 | **1** | 3,145,000 | 221,000 | 66,400 |
|  | **2** | 2,503,000 | 255,000 | 122,000 | **2** | 2,857,000 | 177,000 | 0.0 |
|  | **3** | 1,351,000 | 227,000 | 205,000 | **3** | 1,268,000 | 94,100 | 44,300 |
|  | **4** | 1,561,000 | 161,000 | 133,000 | **4** | 958,000 | 11,100 | 0.0 |
|  | **5** | 1,777,000 | 127,000 | 99,700 | **5** | 2,769,000 | 244,000 | 55400 |
|  |  |  |  |  | **6** | 6,971,000 | 133,000 | 49,800 |
|  | **CV%** | 29.23 | 38.95 | 48.16 | **CV%** | 71.67 | 58.83 | 80.08 |
| **Donor C** | **1** | 2,547,000 | 266,000 | 77,500 | **1** | 3,721,000 | 266,000 | 66,400 |
|  | **2** | 3,367,000 | 487,000 | 88,600 | **2** | 7,265,000 | 443,000 | 0.0 |
|  | **3** | 2,126,000 | 133,000 | 22,100 | **3** | 2,547,000 | 266,000 | 77,500 |
|  | **4** | 3,101,000 | 244,000 | 155,000 | **4** | 5,493,000 | 620,000 | 88,600 |
|  | **5** | 3,101,000 | 177,000 | 88,600 | **5** | 8,970,000 | 664,000 | 0.0 |
|  |  |  |  |  | **6** | 5,936,000 | 443,000 | 0.0 |
|  | **CV%** | 17.63 | 52.32 | 54.71 | **CV%** | 41.16 | 37.49 | 111.03 |
| **Donor D** | **1** | 1,866,000 | 99,700 | 33,200 | **1** | 3,001,000 | 166,000 | 33,200 |
|  | **2** | 1,606,000 | 138,000 | 83,100 | **2** | 2,647,000 | 177,000 | 33,200 |
|  | **3** | 1,561,000 | 88,600 | 83,100 | **3** | 1,866,000 | 99,700 | 33,200 |
|  | **4** | 1,262,000 | 127,000 | 88,600 | **4** | 1,938,000 | 293,000 | 338,000 |
|  | **5** | 2,093,000 | 144,000 | 99,700 | **5** | 2,071,000 | 99,700 | 55,400 |
|  |  |  |  |  | **6** | 2,746,000 | 255,000 | 11,100 |
|  | **CV%** | 18.84 | 20.27 | 33.14 | **CV%** | 20.13 | 43.66 | 149.03 |

**Supplementary Table S8** EV Array co-localization counts for four donors (A-D), calculated as the mean intensity of EV signals obtained from two reciprocal capture–detection pairs. For example, for CD9×CD81, the mean was taken between the intensity of EVs captured with CD9 and detected with CD81, and EVs captured with CD81 and detected with CD9.

|  | **EV Array** | | | | | | | |
| --- | --- | --- | --- | --- | --- | --- | --- | --- |
|  | **D** | CD9∙CD81 | CD9∙CD63 | CD81∙CD63 | **W** | CD9∙CD81 | CD9∙CD63 | CD81∙CD63 |
| **Donor A** | **1** | 5.33 | 0.54 | 0.68 | **1** | 6.44 | 2.94 | 0.60 |
|  | **2** | 5.41 | 2.04 | 0.00 | **2** | 5.25 | 3.06 | 0.00 |
|  | **3** | 5.26 | 1.13 | 0.00 | **3** | 5.33 | 0.54 | 0.68 |
|  | **4** | 2.24 | 0.63 | 0.00 | **4** | 4.25 | 0.65 | 0.00 |
|  | **5** | 3.54 | 0.97 | 0.63 | **5** | 2.04 | 1.50 | 0.00 |
|  |  |  |  |  | **6** | 3.87 | 1.45 | 0.00 |
|  | **CV%** | 32.49 | 56.47 | 137.12 | **CV%** | 33.49 | 64.66 | 155.39 |
| **Donor B** | **1** | 4.20 | 0.77 | 0.00 | **1** | 4.20 | 0.60 | 0.00 |
|  | **2** | 3.03 | 0.58 | 0.00 | **2** | 2.77 | 0.63 | 0.00 |
|  | **3** | 3.23 | 0.00 | 0.00 | **3** | 4.20 | 0.77 | 0.00 |
|  | **4** | 5.10 | 1.38 | 0.00 | **4** | 2.73 | 1.49 | 0.00 |
|  | **5** | 4.19 | 0.67 | 0.00 | **5** | 4.15 | 0.74 | 0.00 |
|  |  |  |  |  | **6** | 5.70 | 1.06 | 0.00 |
|  | **CV%** | 21.19 | 72.49 | NA | **CV%** | 27.92 | 38.32 | NA |
| **Donor C** | **1** | 8.01 | 1.65 | 0.52 | **1** | 5.30 | 0.78 | 0.75 |
|  | **2** | 5.39 | 1.41 | 0.66 | **2** | 5.61 | 2.14 | 0.65 |
|  | **3** | 5.28 | 1.48 | 0.58 | **3** | 8.01 | 1.65 | 0.52 |
|  | **4** | 6.03 | 1.50 | 0.58 | **4** | 6.40 | 2.15 | 0.64 |
|  | **5** | 5.45 | 2.03 | 0.72 | **5** | 5.56 | 1.76 | 0.67 |
|  |  |  |  |  | **6** | 4.60 | 1.69 | 0.73 |
|  | **CV%** | 18.95 | 15.22 | 13.39 | **CV%** | 19.93 | 29.44 | 12.57 |
| **Donor D** | **1** | 7.54 | 1.25 | 0.62 | **1** | 7.77 | 1.32 | 0.62 |
|  | **2** | 11.32 | 1.78 | 0.57 | **2** | 10.90 | 2.34 | 0.57 |
|  | **3** | 12.79 | 1.23 | 0.67 | **3** | 7.54 | 1.25 | 0.62 |
|  | **4** | 7.82 | 0.53 | 0.64 | **4** | 10.81 | 0.89 | 0.60 |
|  | **5** | 8.43 | 0.64 | 0.78 | **5** | 8.47 | 1.27 | 0.63 |
|  |  |  |  |  | **6** | 11.28 | 1.90 | 0.51 |
|  | **CV%** | 24.46 | 46.93 | 11.46 | **CV%** | 18.14 | 35.13 | 7.68 |

**Table S9** Variance components estimated using linear mixed‑effects models for each analyte, partitioned into intra‑individual temporal variability (Timepoint) and residual variance. Variance and corresponding standard deviations (StdDev) are reported for both time scales.

|  |  | **Short-term** | | **Long-term** | |
| --- | --- | --- | --- | --- | --- |
| **Analyte** | **Component** | **Variance** | **StdDev** | **Variance** | **StdDev** |
| **ERYT** | Timepoint | 0.000 | 0.000 | 0.000 | 0.000 |
|  | Residual | 0.001 | 0.037 | 0.001 | 0.038 |
| **PLT** | Timepoint | 0.000 | 0.016 | 0.000 | 0.000 |
|  | Residual | 0.001 | 0.031 | 0.007 | 0.082 |
| **LEUK** | Timepoint | 0.000 | 0.000 | 0.000 | 0.000 |
|  | Residual | 0.016 | 0.126 | 0.031 | 0.176 |
| **LYMPH** | Timepoint | 0.000 | 0.000 | 0.001 | 0.026 |
|  | Residual | 0.006 | 0.081 | 0.010 | 0.100 |
| **MONO** | Timepoint | 0.000 | 0.000 | 0.000 | 0.000 |
|  | Residual | 0.013 | 0.113 | 0.021 | 0.146 |
| **NEUT** | Timepoint | 0.000 | 0.000 | 0.000 | 0.000 |
|  | Residual | 0.034 | 0.185 | 0.061 | 0.246 |
| **EO** | Timepoint | 0.000 | 0.000 | 0.000 | 0.000 |
|  | Residual | 0.299 | 0.547 | 0.045 | 0.212 |
| **BASO** | Timepoint | 0.042 | 0.205 | 0.000 | 0.000 |
|  | Residual | 0.052 | 0.229 | 0.074 | 0.271 |
| **NTA number** | Timepoint | 0.018 | 0.134 | 0.000 | 0.000 |
|  | Residual | 0.173 | 0.415 | 0.132 | 0.363 |
| **NTA size** | Timepoint | 0.001 | 0.023 | 0.000 | 0.000 |
|  | Residual | 0.003 | 0.051 | 0.004 | 0.063 |
| **hFCM CD9** | Timepoint | 0.007 | 0.084 | 0.015 | 0.122 |
|  | Residual | 0.120 | 0.347 | 0.113 | 0.337 |
| **hFCM CD63** | Timepoint | 0.008 | 0.087 | 0.232 | 0.482 |
|  | Residual | 0.178 | 0.422 | 0.643 | 0.802 |
| **hFCM CD81** | Timepoint | 0.063 | 0.251 | 0.072 | 0.269 |
|  | Residual | 0.181 | 0.426 | 0.207 | 0.455 |
| **EV Array CD9** | Timepoint | 0.000 | 0.000 | 0.000 | 0.000 |
|  | Residual | 0.085 | 0.291 | 0.101 | 0.317 |
| **EV Array CD63** | Timepoint | 0.000 | 0.000 | 0.000 | 0.000 |
|  | Residual | 0.049 | 0.221 | 0.132 | 0.363 |
| **EV Array CD81** | Timepoint | 0.009 | 0.096 | 0.007 | 0.083 |
|  | Residual | 0.157 | 0.396 | 0.154 | 0.393 |

**Supplementary Figures**

**Supplementary Figure S1** Light scatter/size calibration. **A)** and **B)** shows the Rosetta calibration settings used for size calibration of data. Flow cytometer settings: Apogee A60-Micro; SSC (405 nm). Rosetta calibration kit CAL003 (beads were run during the same period and using the same settings as when running the samples. Values for sample calculation parameters for EVs in PBS as recommended by manufacturer).

**A**


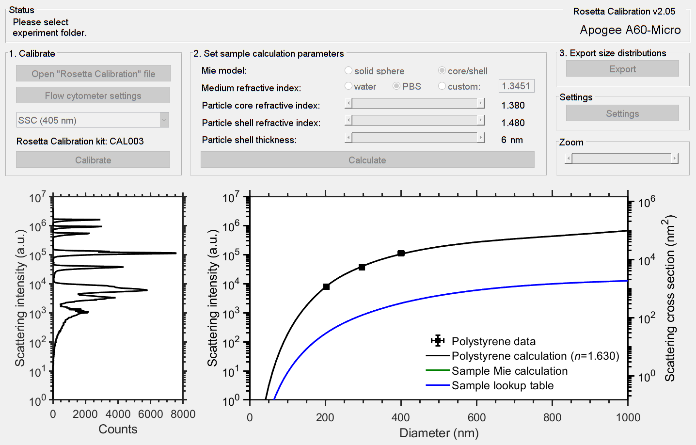


**B**
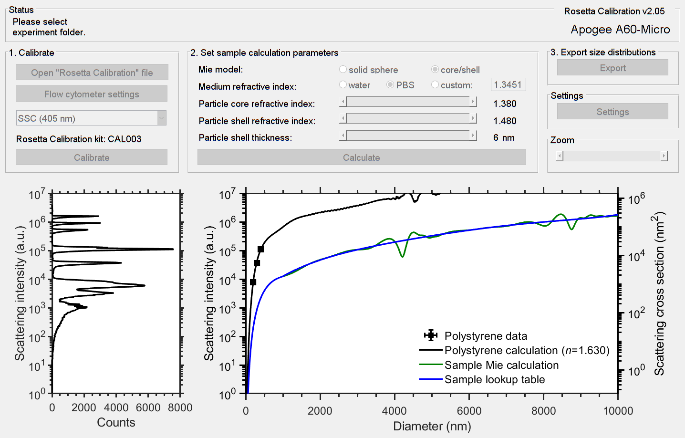


**Supplementary Figure S2** MESF values were calculated via cross-calibration to Rainbow 6-Peak beads (RCP-30-5) in the three channels: 488Grn (FITC), 488Org (PE), and 638Red (APC). **A)** Median Fluorescence Intensity (MFI) of each peak was measured and converted to MESF values. Calibration graphs and linear regression are shown for each channel, log MESF (x-axis) and log MFI (y-axis). **B)** MFI and corresponding MESF values (average of all stained samples) for the three EV-populations: FITC-CD9 (CD9^+^-EVs), PE-CD81 (CD81^+^-EVs), and APC-CD63 (CD63^+^-EVs) according to all three gating strategies.

| **FITC** | | | | |
| --- | --- | --- | --- | --- |
| **Peak** | **Specified intensity (MESF)** | **Measured intensity (MFI)** | **Log10 [Specified intensity (MESF)]** | **Log10 [Measured intensity (MFI)]** |
| **P1** | 1159 | 801 | 3.064083436 | 2.903496947 |
| **P2** | 44709 | 18063 | 4.650394956 | 4.25677786 |
| **P3** | 147552 | 56704 | 5.168945101 | 4.753611781 |
| **P4** | 477000 | 176885 | 5.678518379 | 5.247691313 |
| **P5** | 1280000 | 479000 | 6.10720997 | 5.680335513 |
| **P6** | 2800000 | 1004750 | 6.447158031 | 6.002058015 |

**
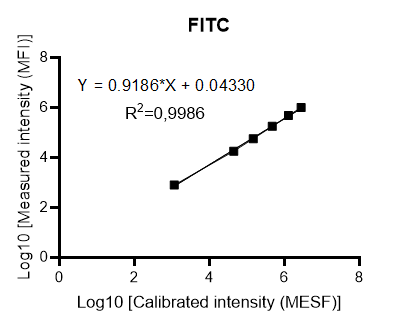
**

| **PE** | | | | |
| --- | --- | --- | --- | --- |
| **Peak** | **Specified intensity (MESF)** | **Measured intensity (MFI)** | **Log10 [Specified intensity (MESF)]** | **Log10 [Measured intensity (MFI)]** |
| **P1** | 45,8 | 425 | 1.660865478 | 2.628644322 |
| **P2** | 2882 | 19847 | 3.459693976 | 4.297683928 |
| **P3** | 10182 | 64690 | 4.007833093 | 4.810833794 |
| **P4** | 35866 | 207332 | 4.554682944 | 5.31666529 |
| **P5** | 109473 | 585625 | 5.03930702 | 5.767619608 |
| **P6** | 264611 | 1327500 | 5.422607894 | 6.12303453 |

**
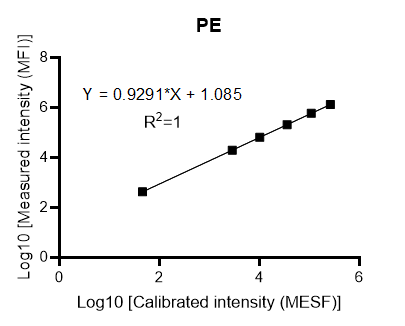
**

| **APC** | | | | |
| --- | --- | --- | --- | --- |
| **Peak** | **Specified intensity (MESF)** | **Measured intensity (MFI)** | **Log10 [Specified intensity (MESF)]** | **Log10 [Measured intensity (MFI)]** |
| **P1** | 2699 | 11445 | 3.431202885 | 4.058601567 |
| **P2** | 6259 | 25329 | 3.796504952 | 4.403615901 |
| **P3** | 15155 | 61397 | 4.180555941 | 4.788146267 |
| **P4** | 42959 | 180810 | 4.633054163 | 5.257223347 |
| **P5** | 107612 | 474750 | 5.031860703 | 5.676464973 |
| **P6** | 177578 | 831000 | 5.24938916 | 5.919601024 |

**
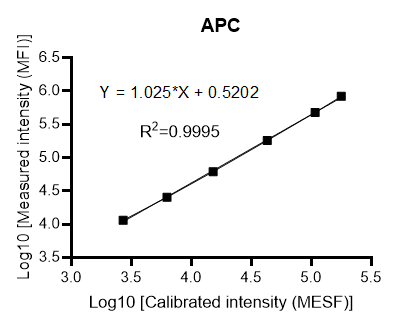
**

**B**

| **EV populations** |  | **MFI** | **MESF** |
| --- | --- | --- | --- |
| **CD9^+^-EVs (0-1000nm)** | CD9^+^ | 1917 | 3485 |
|  | CD9^-^ | 112 | 156 |
|  |  | **MFI** | **MESF** |
| **CD81^+^-EVs (0-1000nm)** | CD81^+^ | 1729 | 213 |
|  | CD81^-^ | 265 | 28 |
|  |  | **MFI** | **MESF** |
| **CD63^+^-EVs (0-1000nm)** | CD63^+^ | 1416 | 367 |
|  | CD63^-^ | 232 | 63 |

**Supplementary Figure S3** Compensation matrix was calculated using Spherotech beads CMIg-08-2K for FITC-CD9, PE-CD81, and APC-CD63 and analysis performed in FlowJo.

| **Channel** | **FITC** | **PE** | **APC** |
| --- | --- | --- | --- |
| **FITC** |  | 14.2 | 6.006 |
| **PE** | 1.6 |  | 4.8 |
| **APC** | 0.6261 | 0.9243 |  |

**Supplementary Figure S4** Gating strategy applied to all samples. **A**) A size gate on 0-1000 nm was applied (after size calibration, histogram). Isotype controls (upper row) were used to define the background for the positive EV-populations, which are depicted (lower row) for the single positive EVs: CD9^+^-, CD81^+^-, and CD63^+^-EVs (highlighted in green boxes), and for double positive populations: CD9^+^CD81^+^-, CD9^+^CD63^+^, and CD81^+^CD63^+^-EVs (highlighted in red boxes). **B**) Size gates for the four sub-analyses: 0-200 nm, 0-1000 nm, 110-200 nm, and 110-1000 nm are depicted as histograms (left column) and dotplots show CD9^+^-EVs (labelled) and included controls (unlabelled, isotype, and detergent) within these size gates as well as aggregate controls for the isotype and antibody mixes (right). Dotplots depicting labelled samples and included controls (unlabelled, isotype, and detergent) within the three size gates as well as aggregate controls for the isotype and antibody mixes (right) for **C**) CD81^+^-EVs, **D**) CD63^+^-EVs, **E**) CD9^+^CD81^+^-EVs, **F**) CD9^+^CD63^+^-EVs, and **G**) CD81^+^CD63^+^-EVs.
 **
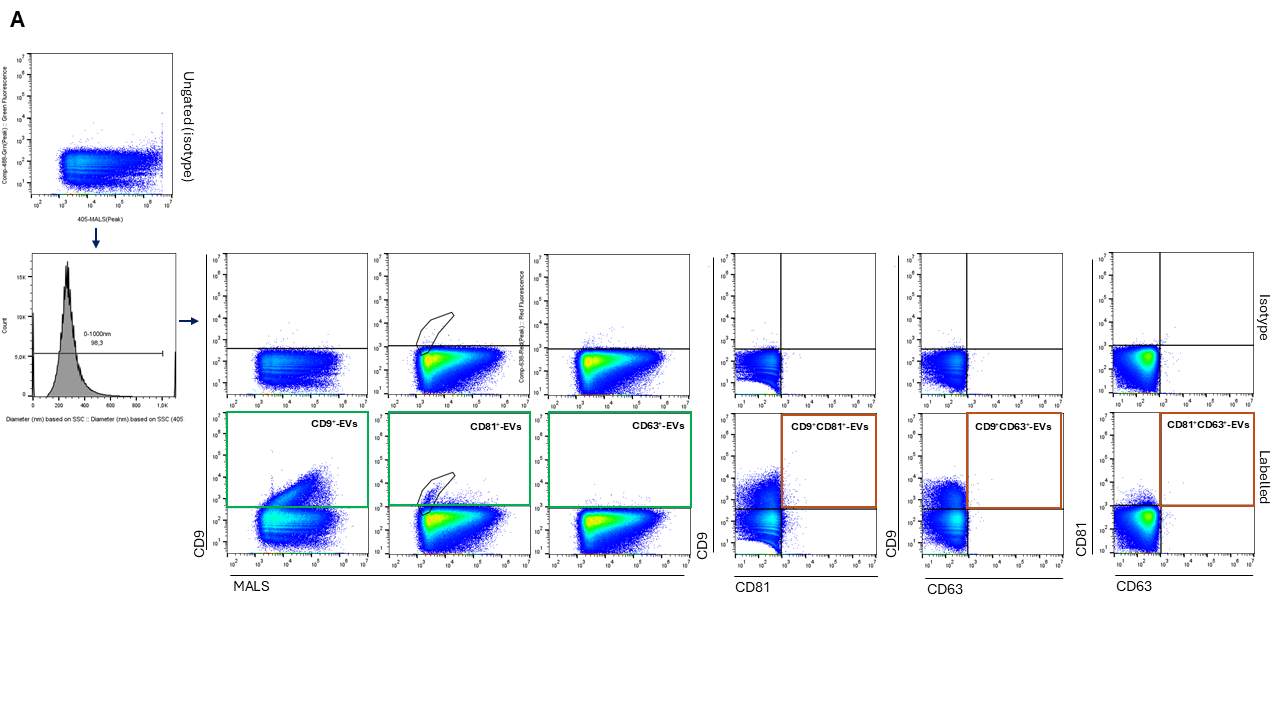

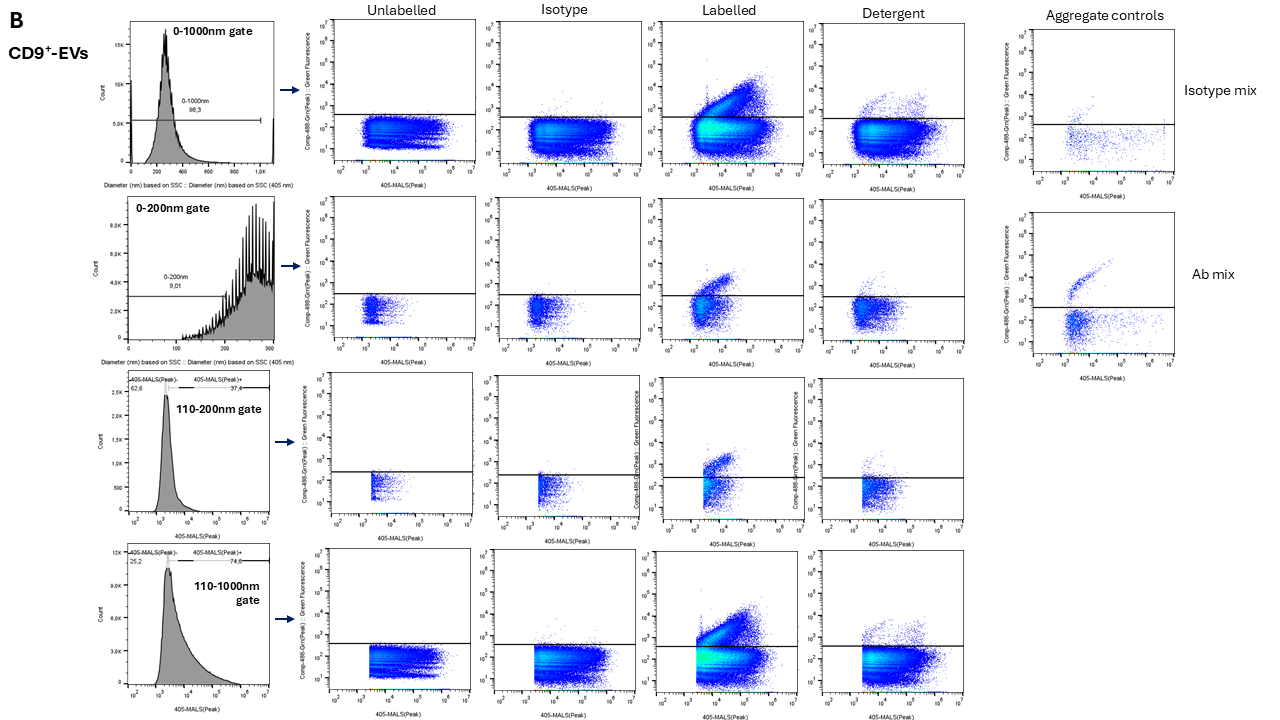

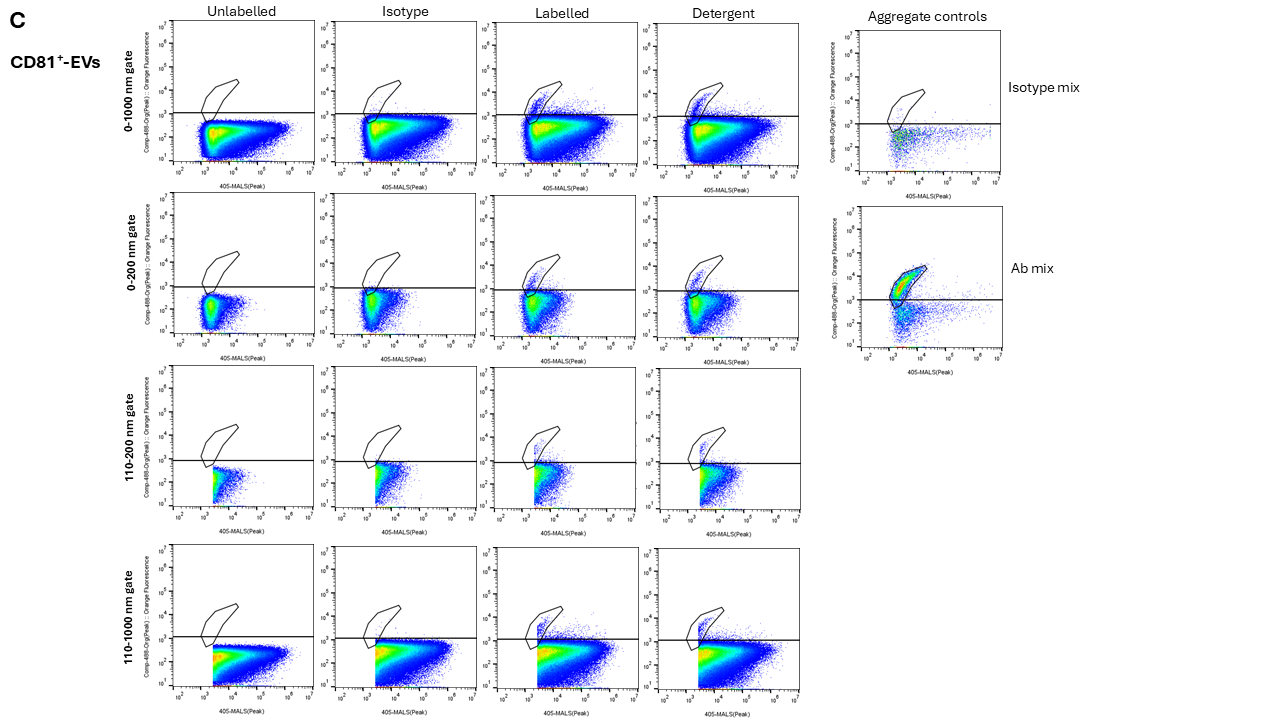

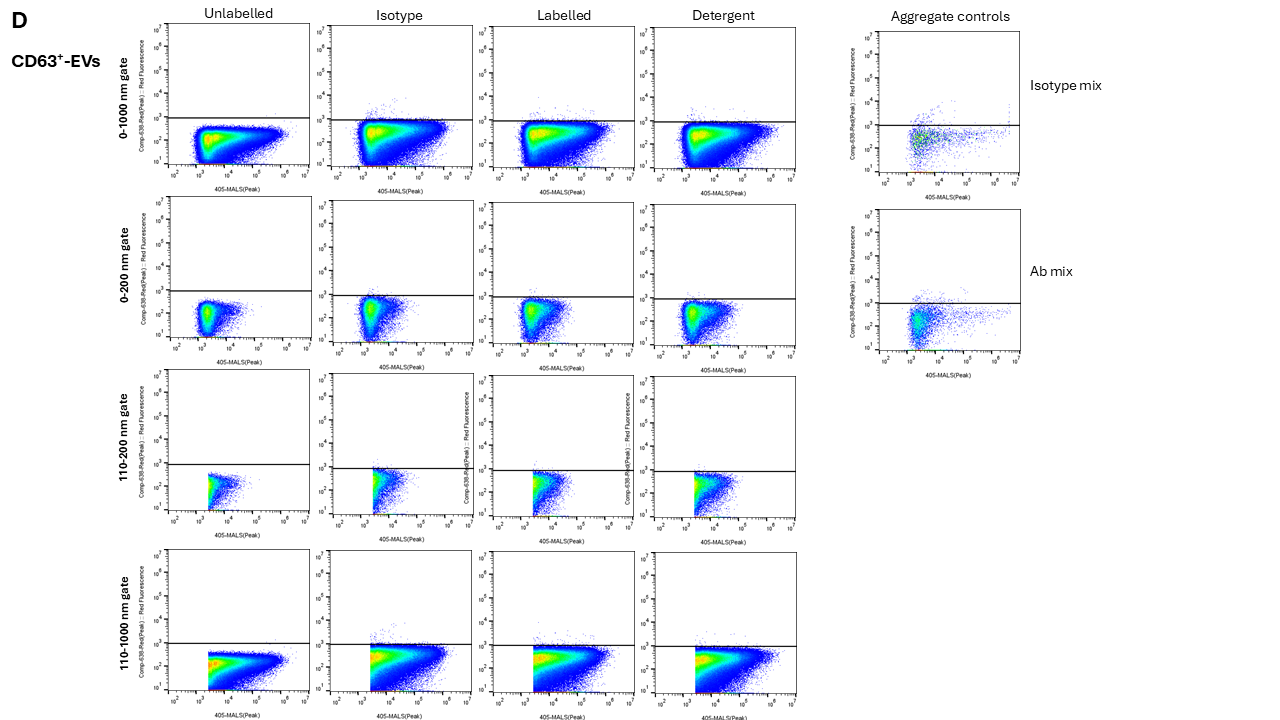

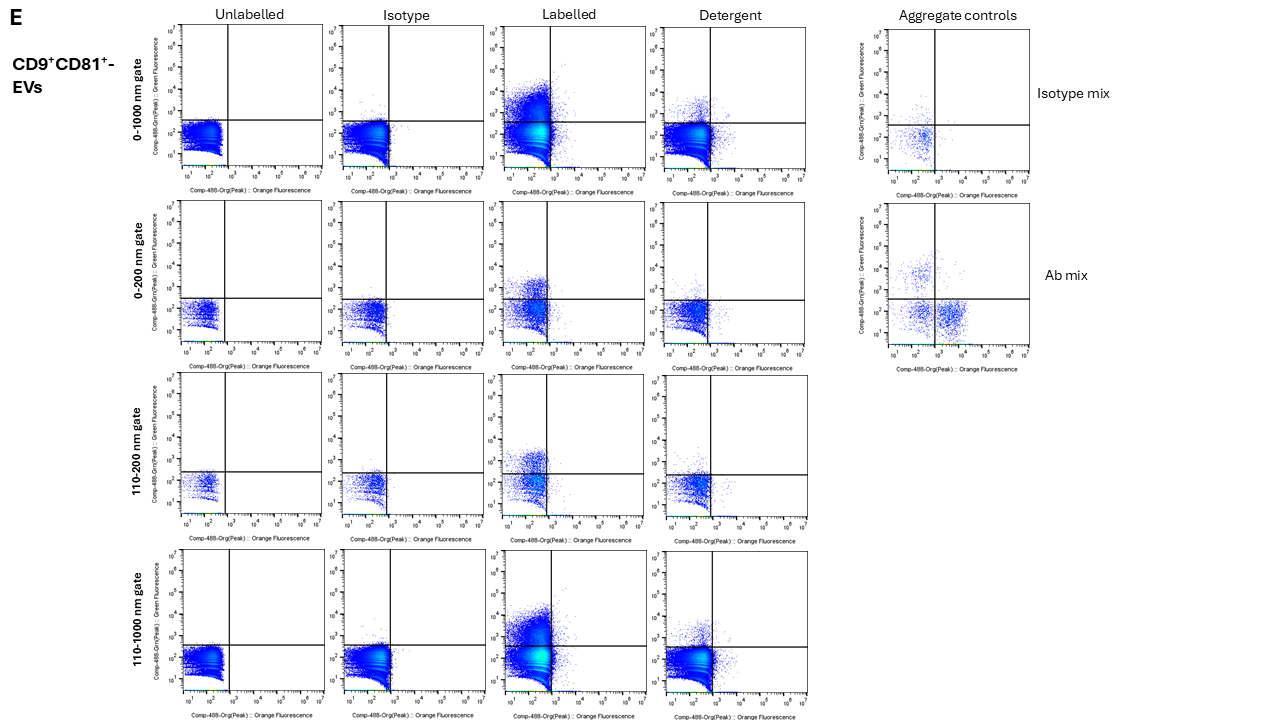

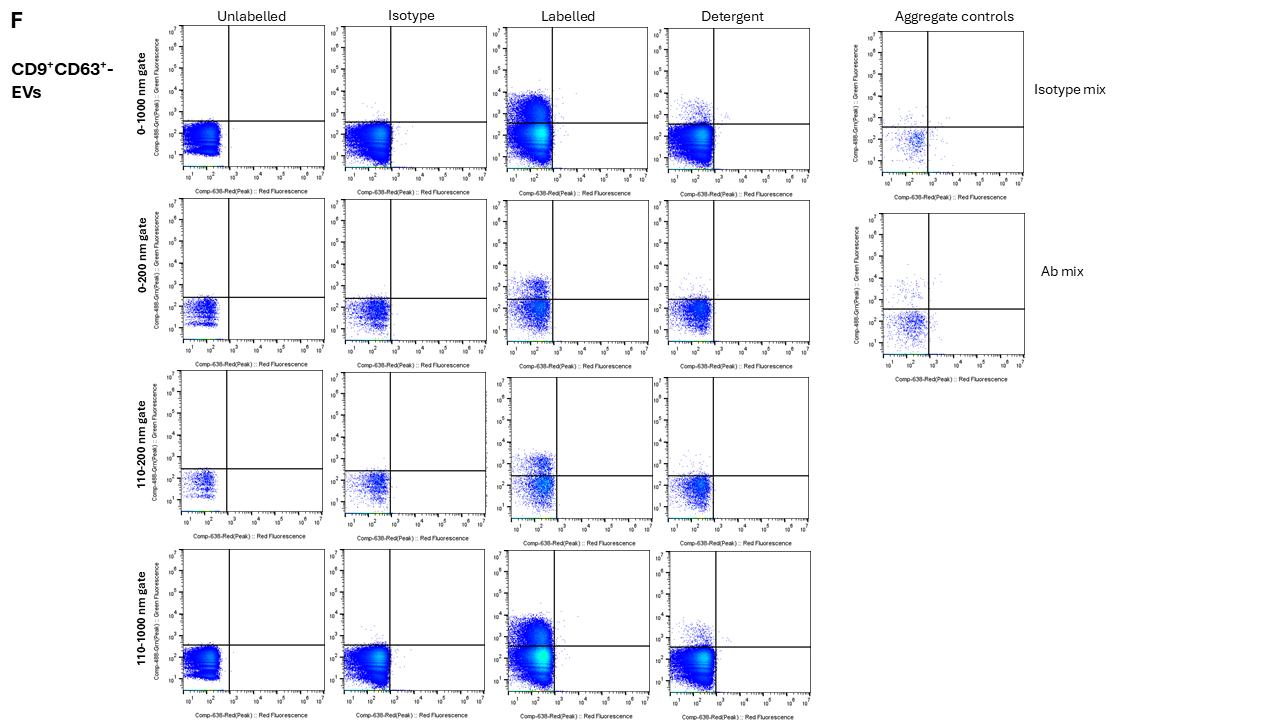

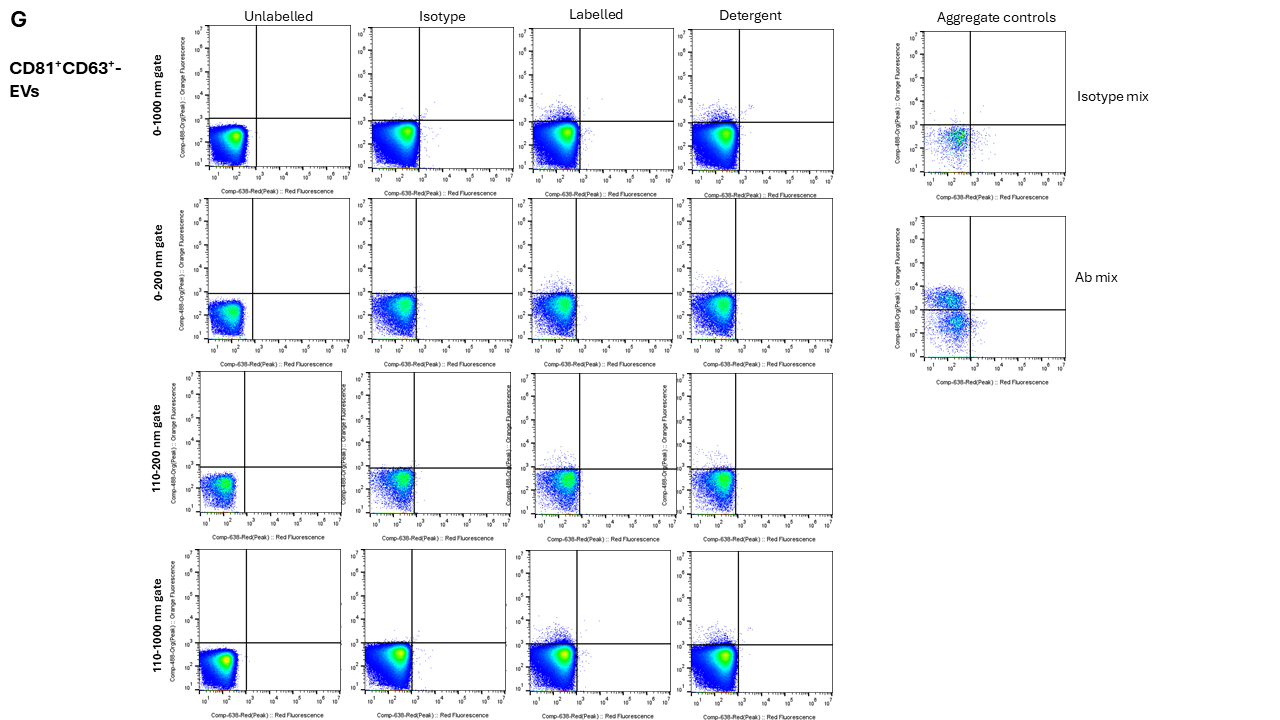
**

**Supplementary Figure S5** Heatmap of intensities measured for four donors (A-D) with EV Array analysis of four donors with 23 capture antibodies and a cocktail of CD9, CD63 and CD81 as detection antibodies.

**
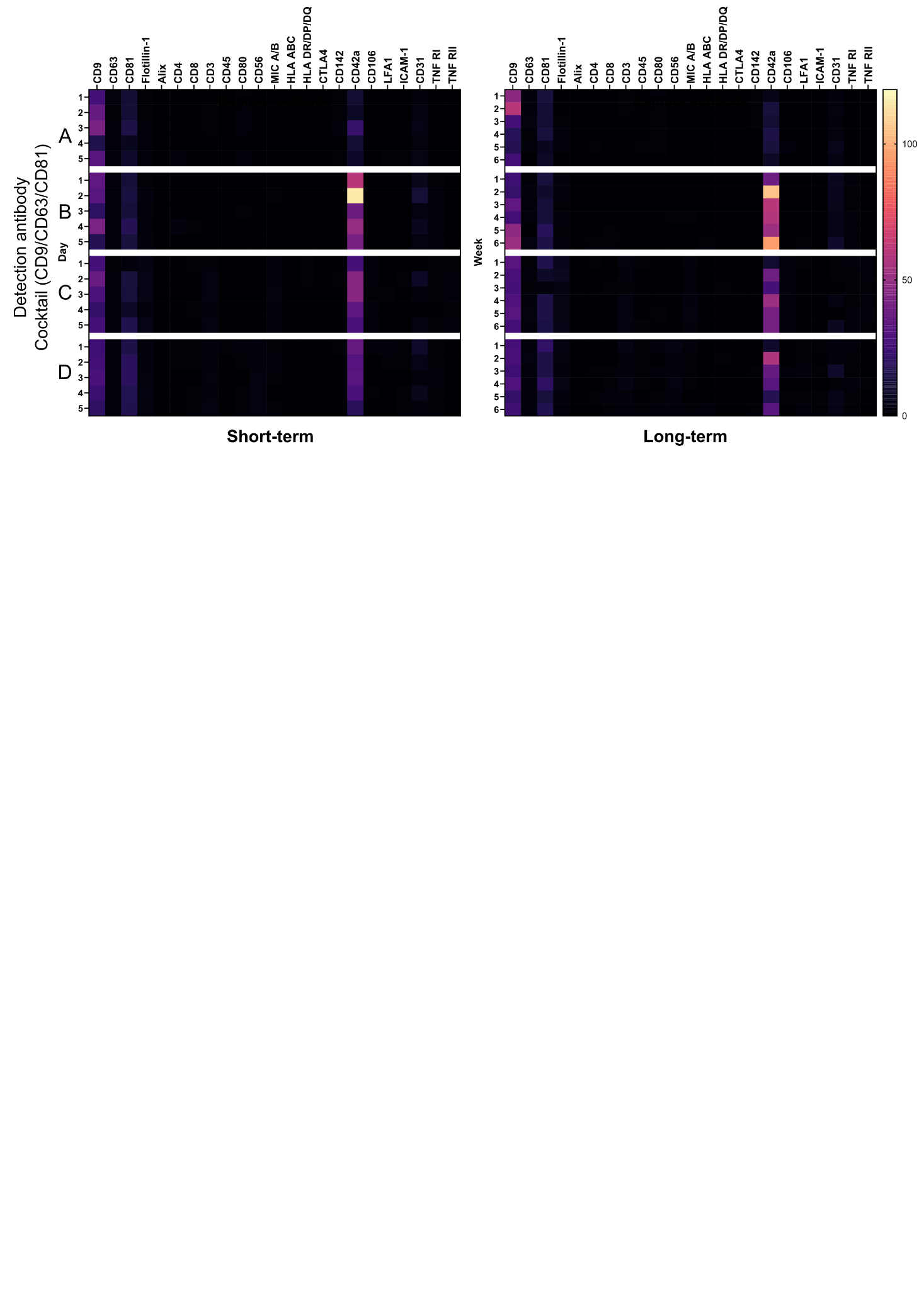
**

**Supplementary Figure S6** Heatmap of intensities measured for four donors (A-D) with EV Array analysis of 23 capture antibodies and a) CD9, b) CD63 and c) CD81 as detection antibodies.**
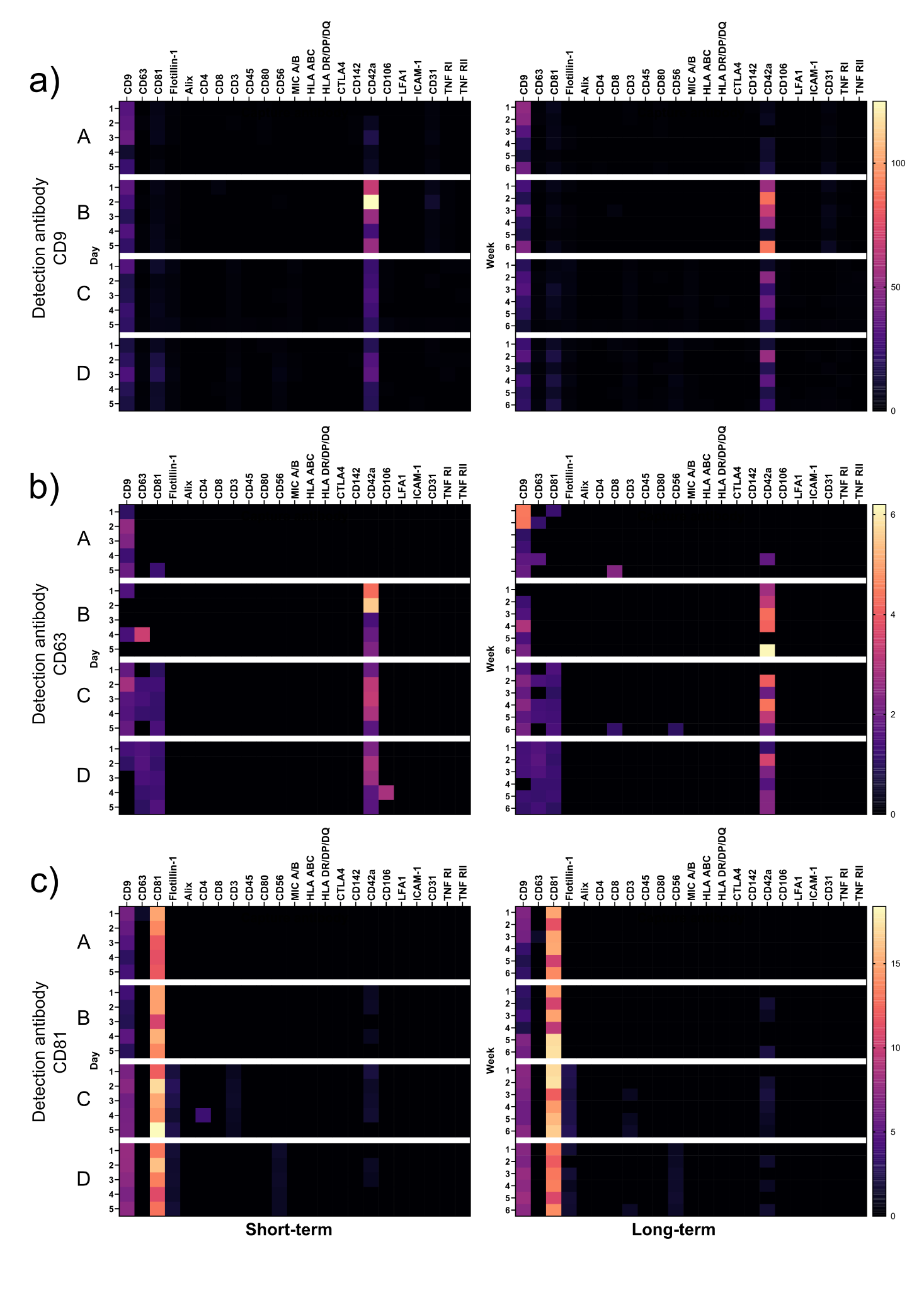
**
